# Assembly and comparative analysis of the first complete mitochondrial genome of *Citrus medica* (Rutaceae)

**DOI:** 10.3389/fpls.2025.1649951

**Published:** 2025-09-18

**Authors:** Xiaoxue Wu, Hong Liu, Siyu Liao, Xinhui Liu, Tao Xie, Lixia Zheng, Yu Hong, Wensheng Chen, Tao Li

**Affiliations:** Department of Horticulture, Foshan University, Foshan, China

**Keywords:** *Citrus medica*, organelle, mitochondrial genome, chloroplast genome, systematic evolution

## Abstract

**Introduction:**

*Citrus medica*, a phylogenetically pivotal progenitor of contemporary citrus cultivars, holds significant economic value due to its medicinal compounds and its role in breeding stress-resistant hybrids. The essential functions of organellar genomes in plant adaptation and metabolic regulation necessitate the characterization of their architecture to elucidate the genomic basis for these traits. However, the absence of a complete mitochondrial genome has hindered investigations into inter-organellar interactions and diversification mechanisms within this key species.

**Methods:**

To address this gap, we used hybrid Illumina-Nanopore sequencing to produce the first mitochondrial genome of *C. medica*. Subsequent annotation and comparative analysis were conducted to identify homologous fragments transferred from the chloroplast genome, repetitive sequences, and RNA editing sites. Phylogenetic reconstruction was performed using 19 mitochondrial genomes, and synteny analysis was employed to assess structural divergence.

**Results:**

The mitochondrial genome of *C. medica* was assembled to a length of 553,930 bp with a GC content of 45.04%, containing 65 genes (36 protein-coding genes, 25 tRNAs, and four rRNAs). Comparative analysis revealed 44 homologous fragments (totaling 41,479 bp) transferred from the chloroplast genome, including two 6,767 bp segments containing 11 genes (*rps7* and 10 tRNAs), with nine tRNA genes being either pseudogenized or lost from the chloroplast genome. The mitogenome includes 633 repetitive sequences (386 dispersed, 215 simple sequence repeats, 32 tandem), with inverted repeats exceeding 11 kb potentially facilitating recombination. Additionally, we predicted 600 RNA editing sites (predominantly C-to-U) in 34 protein-coding genes, affecting amino acid hydrophobicity in 38.83% of these sites. Phylogenetic reconstruction using 19 mitochondrial genomes positioned *C. medica* closest to *C. unshiu*. Synteny analysis highlighted significant structural divergence among Rutaceae mitochondrial genomes, with nucleotide diversity (Pi) indicating 15 polymorphic genes.

**Discussion:**

This study establishes a foundational mitogenomic resource for *C. medica*, demonstrating significant acquisition of chloroplast-derived sequences and dynamic genome architecture, thus advancing the understanding of organellar evolution in citrus and supporting the breeding of stress-resistant cultivars.

## 1 Introduction

*C. medica* (Rutaceae) is an evergreen shrub or small tree characterized by polymorphic fruits that range from sub-ellipsoid to spherical or melon-shaped. The fruits feature oil gland-dotted surfaces with longitudinal ridges that typically culminate in apical papillae, transitioning from green to yellow during ripening (Carvalho et al., 2005). Phytochemical analyses reveal abundant bioactive compounds, including flavonoids (hesperidin, naringin), limonoids (limonin, nomilin), volatile oils (predominantly limonene and citral), vitamin C, pectin, and polysaccharides. Flavonoids exhibit antioxidant, hypoglycemic, and hypolipidemic activities (Sah et al., 2011; Chhikara et al., 2018). Pectin serves as dietary fiber, regulating intestinal functions, while various fruit components exhibit diverse pharmacological effects: the pericarp possesses stomachic and expectorant properties, seeds exhibit antiparasitic activity, and leaf extracts show sedative effects (Chhikara et al., 2018). Beyond their medicinal applications, *C. medica* is widely used in food industries (e.g., jams, preserves, beverages), cosmetic formulations (e.g., essential oils), and religious-cultural practices (Krug, 1943).

Native to Southeast Asia, natural populations of *C. medica* are concentrated in regions such as Yunnan and Tibet (China), as well as India, with cultivated specimens distributed across global tropical and subtropical areas (Wu et al., 2018; Liu et al., 2021; Wang et al., 2025a). As a prominent cultivated citrus variety, it represents one of the largest citrus cultivars worldwide and serves as the progenitor of commercially significant acidic citrus varieties (Wu et al., 2018; Liu et al., 2021; Wang et al., 2025a). Recent studies identify *C. medica*, *C. maxima*, and *C. reticulata* as the three ancestral species of the *Citrus* genus (Wu et al., 2018; Huang et al., 2023). Commercially valuable hybrids, including limes, lemons, sweet oranges, and grapefruits, originated through intraspecific, interspecific, and intergeneric hybridization among these ancestral species, whose unique structural configurations influence the characteristic fruit morphology of modern citrus varieties (Wu et al., 2018; Huang et al., 2023). However, the evolutionary relationships between species and the mechanisms underlying intraspecific diversification remain poorly understood. Mitochondria and chloroplasts, as crucial semi-autonomous organelles within plant cells, are instrumental in elucidating genetic diversity, adaptive evolution, and metabolic regulatory networks through investigations of their genome architecture, functional mechanisms, and evolutionary dynamics (Shen et al., 2022).

Plant mitochondrial genomes exhibit remarkable structural complexity, characterized by expansive sizes, abundant repetitive sequences, and frequent horizontal gene transfer events (Wang et al., 2024; Wang et al., 2025b; Li et al., 2025). These distinctive features are essential for energy metabolism, stress responses, and cytoplasmic male sterility, and they serve as valuable tools for phylogenetic reconstruction and population genetic analyses (Bentolila et al., 2002; Shen et al., 2022). Mitochondrial-plastid DNAs (MTPTs) are a key driver of genomic diversity, with plastid-derived fragments (e.g., tRNA and ribosomal genes) constituting 2.02-5.53% of mitogenomes, as documented in *Ilex rotunda* and *Michelia*, where intact tRNA genes (trnD-GUC, trnW-CCA) retain functionality after transfer​​ (Wang et al., 2024, 2025b). In contrast, chloroplast genomes demonstrate higher evolutionary conservation while providing critical insights into plant evolution and facilitating inter-organellar gene transfer with mitochondrial genomes (Gandini et al., 2019; Ma et al., 2022; Lu et al., 2023). The cross-organellar transfer of tRNA and ribosomal genes reveals dynamic interactions between organelles and their co-evolutionary trajectories (Yang et al., 2023).

As an ancestral *Citrus* species with unique medicinal properties and stress-resistant characteristics, *C. medica* remains genomically under characterized, particularly regarding its complete mitochondrial genome. This study is the first to employ a hybrid assembly strategy integrating Illumina and Nanopore sequencing technologies for the *de novo* assembly and functional annotation of the *C. medica* mitochondrial genome. Through comprehensive characterization of structural features, repetitive element distribution, and RNA editing patterns, coupled with comparative genomic analysis of chloroplast-mitochondrial DNA transfer mechanisms, this study establishes a theoretical foundation for understanding Citrus evolution, organelle interaction networks, and functional gene discovery. The results pave the way for the development of stress-resistant citrus cultivars and molecular markers while providing novel perspectives on mitochondrial genome plasticity and its significance in plant evolution and crop improvement.

## 2 Materials and methods

### 2.1 Plant materials and preparations of samples

*C. medica* samples were collected from the greenhouse at Foshan University in Foshan City, Guangdong Province, China (N23.02, E113.14), which originated from germplasm resources in Metuo County, Xizang Province, China (N29.33, E95.33). After surface disinfection of fresh leaves using 75% alcohol, DNA extraction was conducted using the OMEGA Plant DNA Kit D2485-04 (OMEGA Bio-Tek Co., Guangdong, China) following the manufacturer’s instructions. The quality and concentration of the extracted DNA were assessed using a Qubit 2.0 (Thermo Fisher Scientific Inc., Waltham, MA, USA) and an Agilent 2100 (Agilent Technologies Inc., Santa Clara, CA). The extracted DNA was stored at -80 °C for preservation and used as sequencing material for subsequent steps involving the genomes of plant organelles.

### 2.2 Sequencing and database analyses

Short-read sequencing of the *C. medica* organelle genomes (mitochondria and chloroplast) was performed using the Illumina NovaSeq X Plus whole-genome resequencing platform. The fastp software was employed for filtering and quality control of raw short-read sequencing data to obtain high-quality sequencing datasets (Chen et al., 2018). For long-read sequencing, the Nanopore PromethION sequencer (Oxford Nanopore Technologies, Oxford, UK) platform was used. The NanoPack software was used to perform data filtering and quality control on the raw long-read sequencing data (De Coster et al., 2018).

### 2.3 Organelle genome assembly, annotation, and structure prediction

Multiple sophisticated methods were implemented for the assembly of the *C. medica* mitochondrial genome. Initially, the mitochondrial genome of *C. sinensis* (GenBank accession number: NC_037463.1) served as the reference genome. Bowtie2 (version 2.3.5.1) and minimap2 were employed to map the high-quality short-read and long-read sequencing data, acquired after rigorous filtration and quality control procedures, back onto the reference genome (Langmead and Salzberg, 2012; Li, 2021). Subsequently, the Unicycler software (version 0.4.8), using default parameters, was employed to perform the assembly operation on the aligned data (Wick et al., 2017). The GetOrganelle software was used for chloroplast genome assembly (Jin et al., 2020). The accuracy and integrity of the assembled organelle genomes were confirmed using CLC Genomics Workbench 23 (Qiagen Bioinformatics, Aarhus, Denmark). Annotation of the *C. medica* mitochondrial genome was performed using MGA (http://www.1kmpg.cn/ipmga/), with subsequent manual adjustments based on the mitochondrial genome of *C. sinensis* (GenBank accession number: NC_037463.1). The OGDRAW software was used for the plotting of the mitochondrial genome map (Greiner et al., 2019). The CPGAVAS2 tool was used to annotate the chloroplast genome of *C. medica* based on the reference genome of *C. sinensis* (GenBank accession number: NC_008334.1) (Shi et al., 2019). Annotated maps of the chloroplast genome and cis-splicing/trans-splicing genes were generated using CPGView (Liu et al., 2023).

RNA editing sites were predicted using the Deepred-Mt website (http://47.96.249.172:16084/deepredmt.html) (Edera et al., 2021), and sites with scores higher than 0.2 were screened. After extracting total RNA from mature leaves of *C. medica*, RNA was fragmented with ion reagents and bound to random primers. First-strand cDNA was synthesized via reverse transcription, followed by second-strand cDNA synthesis using residual RNA as primers after RNA cleavage in hybrids. Double-stranded cDNA undergoes end repair, dA-tailing, and ligation with Y-shaped adapters. Adapter self-ligated fragments are removed by magnetic beads, with library templates enriched by PCR and recovered via magnetic beads (Oligo (dT)). The library is then sequenced using the NovaSeq 6000 Illumina HiSeq platform. To identify potential RNA editing sites, these RNA-seq data were analyzed using a reference-based variant detection approach. ​​First​​, raw reads were aligned to CDS of the reference genome in local alignment mode using Bowtie2 (v2.3.5.1) (Langmead and Salzberg, 2012). ​​The resulting alignments​​ were sorted and indexed using Samtools (v1.9), ​​followed by filtering​​ to retain reads with a minimum mapping quality (MAPQ) ≥ 40 (Danecek et al., 2021). ​​Subsequently​​, variant calling was performed on the filtered alignments using Bcftools (v1.9) (Danecek et al., 2021) to identify discordant single-nucleotide variants (SNVs) relative to the reference genome. ​​Finally​​, putative variants were filtered to retain only those supported by a minimum sequencing depth of 3, designating the remaining loci as candidate RNA editing sites. The BLASTN software was employed to align homologous sequences between the chloroplast and mitochondrial genomes of *C. medica* (Chen et al., 2015), with the E-value set to 1e^-5^ and other parameters maintained at their default settings. The Circos package (version 0.69-5) was used to visualize the results (Zhang et al., 2013).

### 2.4 Phylogenetic evolution and sequence collinearity

Nineteen complete mitochondrial genome sequences from two distinct orders (Sapindales and Aquifoliales) were obtained from the National Center for Biotechnology Information (NCBI) database, including those of *C. sinensis* (NC_037463.1), *C. reticulata* (NC_086688.1), *C. maxima* (PP035765.1), *C. unshiu* (NC_057142.1), *Phellodendron amurense* (PP492704.1, PP492705.1), *Sapindus mukorossi* (NC_050850.1), *Litchi chinensis* (PP932631.1), *Nephelium lappaceum* (PP916047.1), *Xanthoceras sorbifolium* (MK333231.1), *Acer yangbiense* (NC_059858.1), *Acer miaotaiense* (MZ636518.1), *Acer truncatum* (MZ318049.1), *Mangifera longipes* (NC_060990.1), *Mangifera sylvatica* (MZ751077.1), *Mangifera persiciforma* (MZ751076.1), *Ilex micrococca* (PP994859.1), *Ilex metabaptista* (NC_081509.1), and *Ilex pubescens* (NC_045078.1). Species from the genus *Ilex* (*I. micrococca* (PP994859.1), *I. metabaptista* (NC_081509.1), and *I. pubescens* (NC_045078.1)) were designated as the outgroup. *Ilex* species (order Aquifoliales) were chosen for this role because Aquifoliales belongs to the asterid clade, which—together with the rosid clade—constitutes the core Pentapetalae. Given that the ingroup taxa in this study (Sapindales) are classified within the rosids, this deep phylogenetic divergence between the asterid and rosid lineages ensures that *Ilex* provides a robust and evolutionarily distant root for resolving phylogenetic relationships within Sapindales. The mitochondrial genome of *C. medica*, obtained through sequencing and assembly in this research, was incorporated to construct a phylogenetic tree for in-depth phylogenetic analysis.

During the construction of the phylogenetic tree, MAFFT software (version 7.427) was used to perform multiple sequence alignment on the gene sequences encoded by the 20 mitochondrial genomes (Katoh and Standley, 2013). The trimAl software was used for trimming (parameters: -gt 0.7) (Capella-Gutiérrez et al., 2009). After trimming, the jModelTest software was employed for model prediction (Posada, 2008). Using RAxML software (version 8.2.10), the GTRGAMMA model was selected with 1000 bootstrap replications to construct the maximum likelihood phylogenetic tree (Stamatakis, 2014). The ITOL software (version 4.0) was then used to visualize the maximum likelihood tree (Letunic and Bork, 2019).

The BLASTN algorithm was used to compare the mitochondrial genome of *C. medica* with those of five related species within Rutaceae previously reported. This comparison aimed to identify homologous sequences among these six mitochondrial genomes, with parameter settings configured as -evalue 1e^-5^ and -word_size 7. The Multiple Synteny Plot plugin within TBtools software was then employed to visualize homologous sequences of no less than 300 bp (Chen et al., 2020).

### 2.5 Analysis of codon usage bias, repetitive sequences, and nucleotide diversity

Following the extraction of protein-coding sequences from the genome using TBtools software, the codonW software was employed for the analysis of codon usage bias of the protein-coding genes (PCGs) within the mitochondrial genome of *C. medica* using default parameters (Sharp and Li, 1986).

To analyze repetitive sequences within the mitochondrial genome of *C. medica*, the MISA web tool (https://webblast.ipk-gatersleben.de/misa/index.php?action=1) (version 2.1, parameter settings: 1-10 2-5 3-4 4-3 5-3 6-3) was used to identify simple sequence repeat (SSR) sequences (Beier et al., 2017). Tandem repeats were identified using the Tandem Repeats Finder software (parameter settings: 2 7 7 80 10 50 2000 -f -d -m) (Benson, 1999). Dispersed repeats were identified using the Vmatch software (version 2.3.0), applying a minimum sequence length of 30 bp and a Hamming distance of 3 (Kurtz, 2010).

For nucleotide polymorphism analysis, MAFFT software (v7.427, under the –auto mode) was employed to conduct global alignments of homologous gene sequences among diverse species (Katoh and Standley, 2013). Subsequently, DnaSP5 was utilized to calculate the Pi value for each individual gene (Librado and Rozas, 2009).

## 3 Results

### 3.1 Organelle genome assembly and gene function annotation

**Figure 1:**
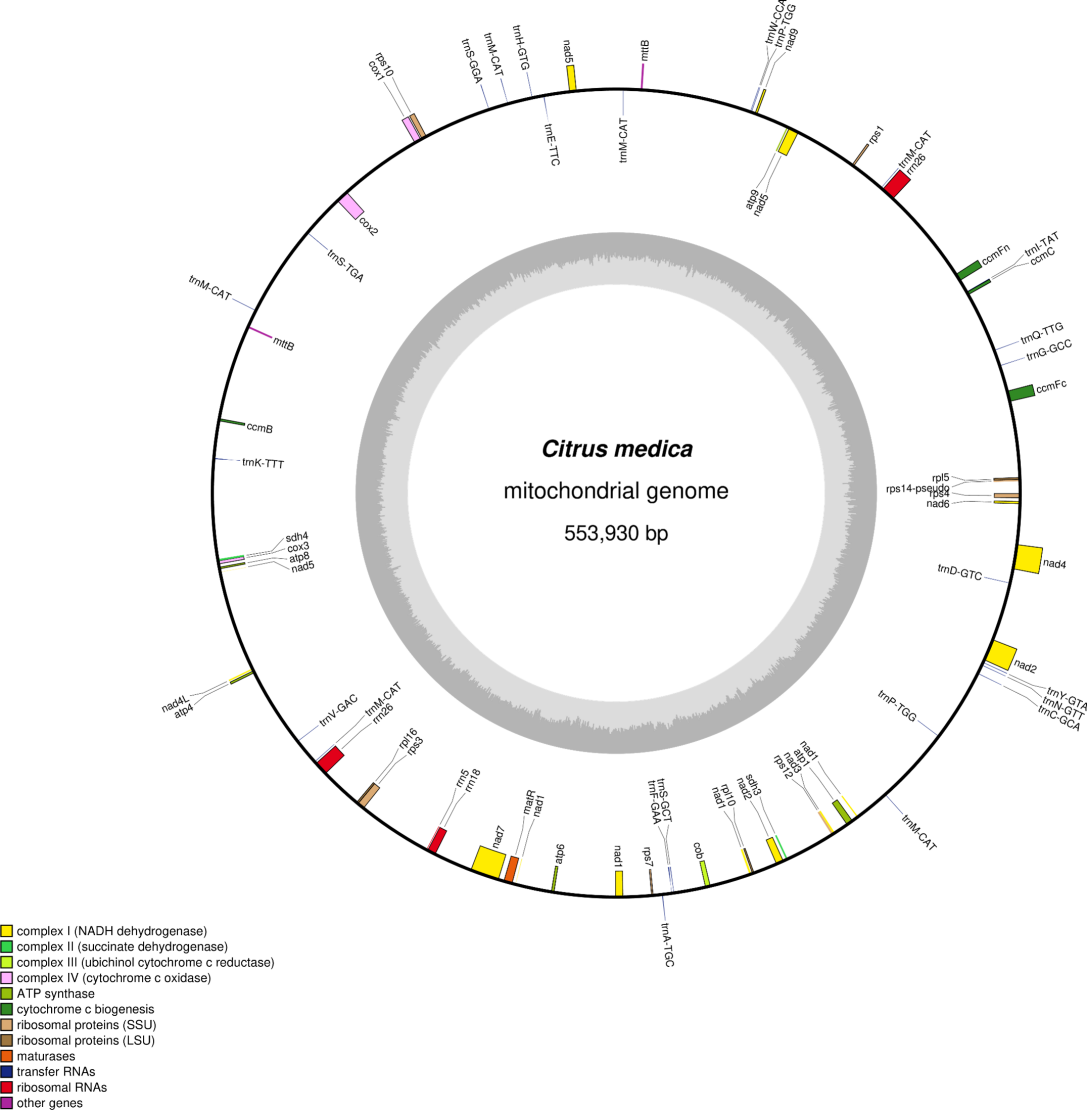
Map of the *C. medica* mitochondrial genome after the double-bifurcation structures were resolved using long-read data.

The mitochondrial genome was assembled using a hybrid strategy that integrated long-read and short-read sequencing data. Due to its complex conformation, characterized by branched architectures and abundant repetitive sequences, long-read sequencing data were employed to verify and resolve these structural complexities, ultimately yielding a linear mitochondrial genome assembly (**Figure 1**). The mitochondrial genome of *C. medica* was found to consist of six ​​assembly contigs with the following lengths: contig 1 (269,363 bp), contig 2 (98,057 bp), contig 3 (87,711 bp), contig 4 (54,040 bp), contig 5 (11,216 bp), and contig 6 (11,189 bp). Notably, contigs 5 and 6 exhibited double-bifurcation structures (**Supplementary Figure S1**), and the connectivity between these two contigs and other genomic regions was validated by Nanopore read alignments spanning full junctions (**Supplementary Figure S2**). The complete mitochondrial genome spanned 553,930 bp with a GC content of 45.04% (GenBank accession number: PQ636878), and its structure was confirmed through long-read sequencing data. Annotation of the mitochondrial genome revealed 65 genes, including five ATP synthase genes (*atp1*, *atp4*, *atp6*, *atp8*, *atp9*), four cytochrome c biogenesis genes (*ccmB*, *ccmC*, *ccmFc*, *ccmFn*), one ubiquinol cytochrome c reductase gene (*cob*), three cytochrome c oxidase genes (*cox1*, *cox2*, *cox3*), one maturase gene (*matR*), two transport membrane protein gene (*mttB* (×2)), nine NADH dehydrogenase genes (*nad1*, *nad2*, *nad3*, *nad4*, *nad4L*, *nad5*, *nad6*, *nad7*, *nad9*), three ribosomal large subunit genes (*rpl10*, *rpl16*, *rpl5*), six ribosomal small subunit genes (*rps1*, *rps10*, *rps12*, *rps3*, *rps4*, *rps7*), two succinate dehydrogenase genes (*sdh3*, *sdh4*), four rRNA genes (*rrn18*, *rrn26* (×2), *rrn5*), and 25 tRNA genes (**Figure 1**; **Table 1**).

**Table 1:** Gene profile of the *C. medica* mitogenome.

Group of genes	Gene name
ATP synthase	*atp1*, *atp4*, *atp6*, *atp8*, *atp9*
Cytochrome c biogenesis	*ccmB*, *ccmC*, *ccmFc*, *ccmFn*
Ubichinol cytochrome c reductase	*cob*
Cytochrome c oxidase	*cox1*, *cox2*, *cox3*
Maturases	*matR*
Transport membrane protein	*mttB* (×2)
NADH dehydrogenase	*nad1*, *nad2*, *nad3*, *nad4*, *nad4L*, *nad5*, *nad6*, *nad7*, *nad9*
Ribosomal large subunit genes	*rpl10*, r*pl16*, *rpl5*
Ribosomal small subunit genes	*rps1, rps10, rps12, rps3, rps4, rps7*
Succinate dehydrogenase	*sdh3*, *sdh4*
Ribosomal RNAs	*rrn18*, *rrn26* (×2), *rrn5*
Transfer RNAs	*trnA-TGC*, *trnC-GCA*, *trnD-GTC*, *trnE-TTC*, *trnF-GAA*, *trnG-GCC*, *trnH-GTG*, *trnI-TAT*, *trnK-TTT*, *trnM-CAT* (×6), *trnN-GTT*, *trnP-TGG* (×2), *trnQ-TTG*, *trnS-GCT*, *trnS-GGA*, *trnS-TGA*, *trnV-GAC*, *trnW-CCA*, *trnY-GTA*

The numbers in parentheses represent the copy number of the gene.

**Figure 2:**
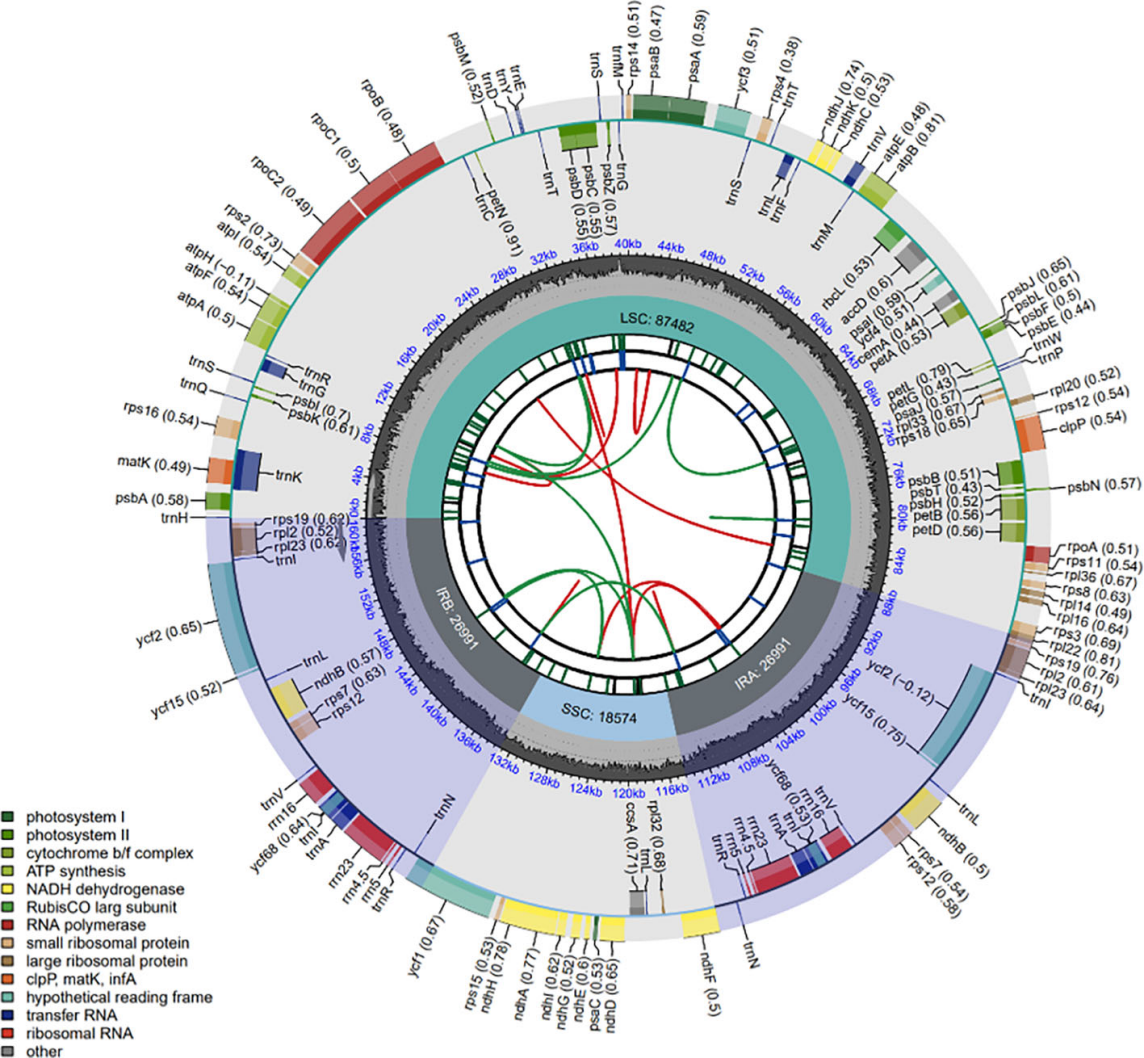
Complete chloroplast genome map of *C. medica.* From the center to the outside, there are six tracks. The first track is dispersed repeats consisting of direct (D) and palindromic (P) repeats, which are connected by red and green arcs. The second track shows long tandem repeats (short blue bars). The third track shows short tandem repeats or microsatellite sequences (short bars with different colors). Small single-copy (SSC), inverted repeat (IRa and IRb), and large single-copy (LSC) areas are displayed in the fourth track. The GC content of the genome is plotted on the fifth track. Shown between the fourth and fifth tracks is the base frequency of each locus in the genome. Gene annotation is displayed in the sixth track. Genes are color-coded according to their functional classification. The transcription directions of inner genes and outer genes are clockwise and anticlockwise, respectively. The functional classification of genes is shown in the lower left corner.

We performed *de novo* assembly of the chloroplast genome for *C. medica* by integrating both next-generation sequencing and third-generation sequencing data. The complete chloroplast genome (GenBank accession number: PP863286) had a length of 160,038 bp, with the nucleotide base composition as follows: G: 18.86%, C: 19.59%, A: 30.47%, T: 31.08%. This genome consists of four regions: an 87,482 bp large single-copy region, an 18,574 bp small single-copy region, and a pair of 26,991 bp inverted repeat regions. In total, the chloroplast genome contains 134 genes, including 89 PCGs, 37 tRNA genes, and eight rRNA genes (**Figure 2**). Among the twelve PCGs containing introns, two genes (*ycf3* and *clpP*) possessed two introns each, while one intron was present in ten genes (*rps16*, *atpF*, *rpoC1*, *petB*, *petD*, *ndhA*, *ndhB* [×2], *rpl2* [×2]) (**Supplementary Figure S3**). Additionally, trans-splicing was detected in the *rps12* gene (**Supplementary Figure S4**).

### 3.2 Comparative homology assessment between mitochondrial and chloroplast genomes of *C. medica*

**Figure 3:**
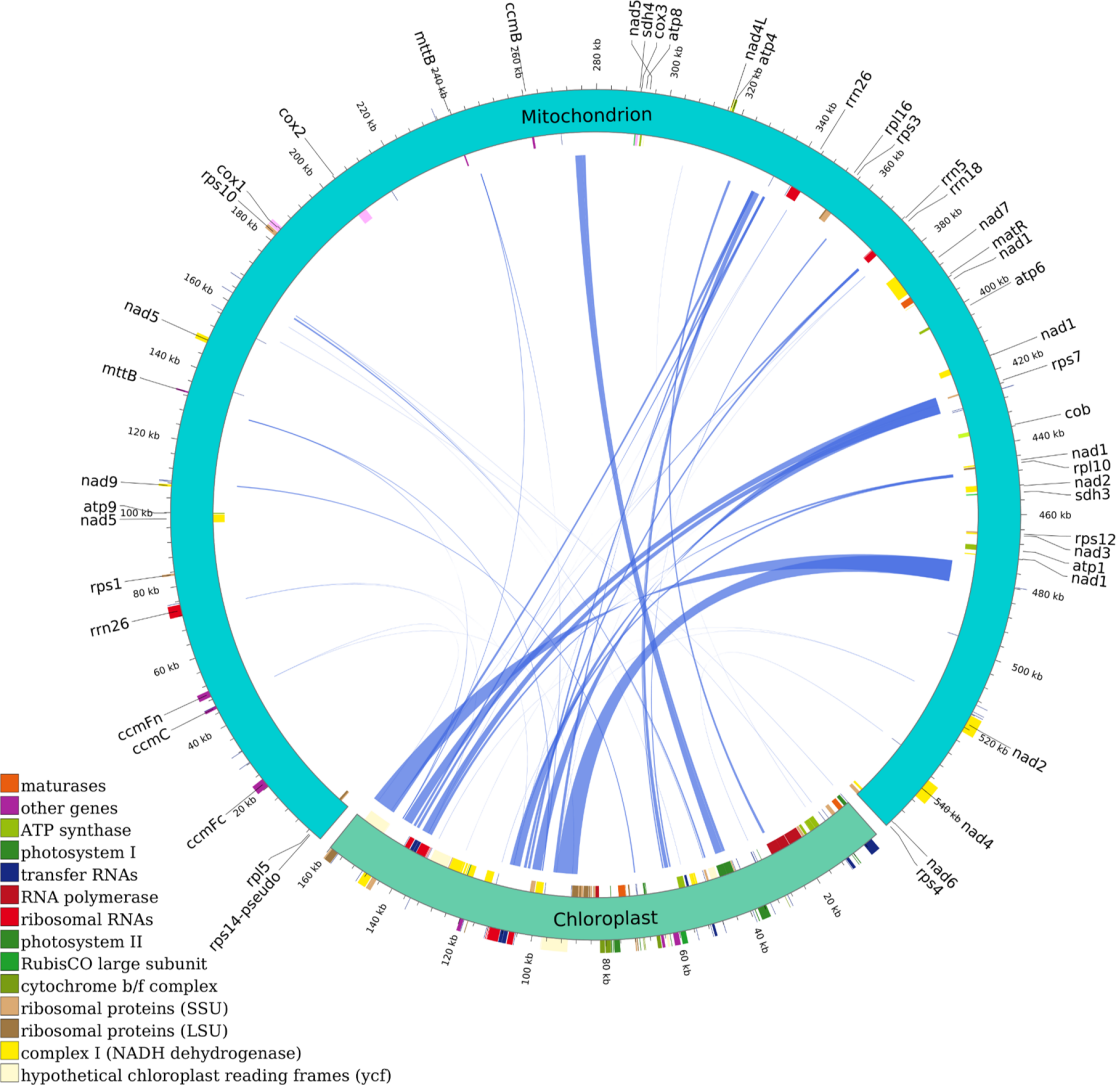
Homologous analysis based on mitochondrial and chloroplast genomes of *C. medica*. The homologous fragments are indicated using the blue lines.

Intercompartmental sequence exchange between mitochondria and chloroplasts is a ubiquitous phenomenon in higher plants, with mitochondrial genome fragments demonstrating conserved homologous sequences within chloroplast genomes. This comparative analysis provides insights into the mechanisms governing horizontal gene transfer in chloroplast genomes, elucidating its evolutionary significance in plant phylogeny. Consequently, we conducted a systematic comparative analysis of mitochondrial and chloroplast genome homology in *C. medica*. Sequence homology analysis identified 44 conserved inter-organellar fragments between the mitochondrial and chloroplast genomes, ranging in size from 40 to 6,767 base pairs (bp), cumulatively spanning 41,479 bp (**Figure 3**; **Supplementary Table S1**).

The two longest homologous segments (CP-1 and CP-2, each 6,767 bp) accounted for 2.44% of the mitochondrial genome, with DNA transfer events predominantly localized to the inverted repeat (IR) regions of the *C. medica* chloroplast genome. Functional annotation of these transferred sequences revealed 11 intact genes, comprising one protein-coding gene (*rps7*) and ten tRNA genes (*trnA-TGC, trnV-GAC, trnP-TGG, trnS-GGA, trnI-TAT, trnN-GTT, trnH-GTG, trnD-GTC, trnW-CCA, trnM-CAT*). Notably, nine tRNA genes (excluding *trnW-CCA*) exhibited complete loss or pseudogenization within the chloroplast genome (**Figure 3**; **Supplementary Table S1**).

### 3.3 Integrative analysis of phylogenetic relationships and syntenic architecture

**Figure 4:**
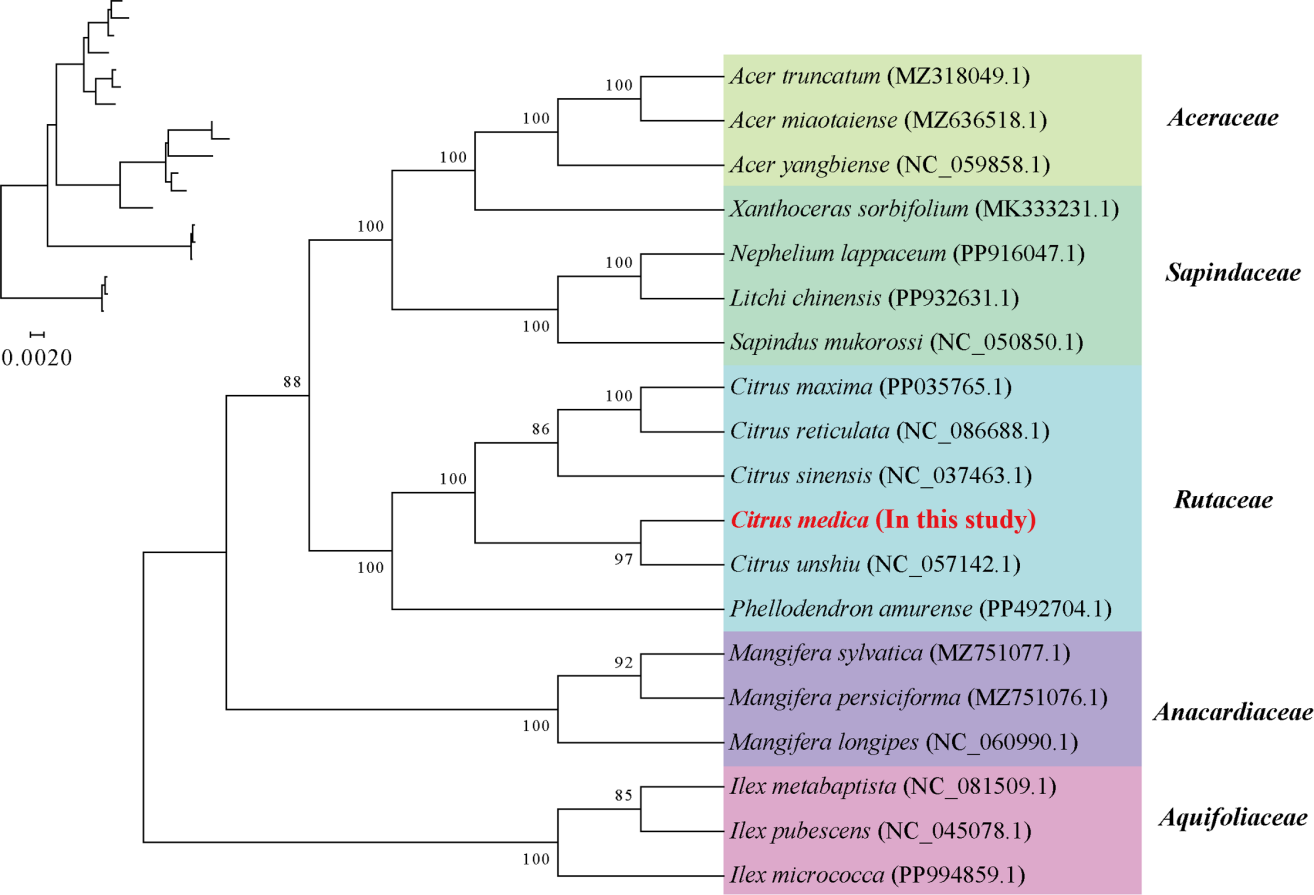
Construction of the maximum likelihood tree based on the mitochondrial genomes of the 19 species. The numbers indicate the bootstrap values for the maximum likelihood (ML) tree.

A maximum likelihood phylogenetic tree was constructed using conserved homologous genes from 19 mitochondrial genomes representing two angiosperm orders (Sapindales and Aquifoliales). Sixteen nodes exhibited bootstrap support values exceeding 80%, with 11 nodes achieving maximal nodal support (BS = 100%). Notably, *C. medica* demonstrated close phylogenetic affinity to *C. unshiu* (**Figure 4**).

**Figure 5:**
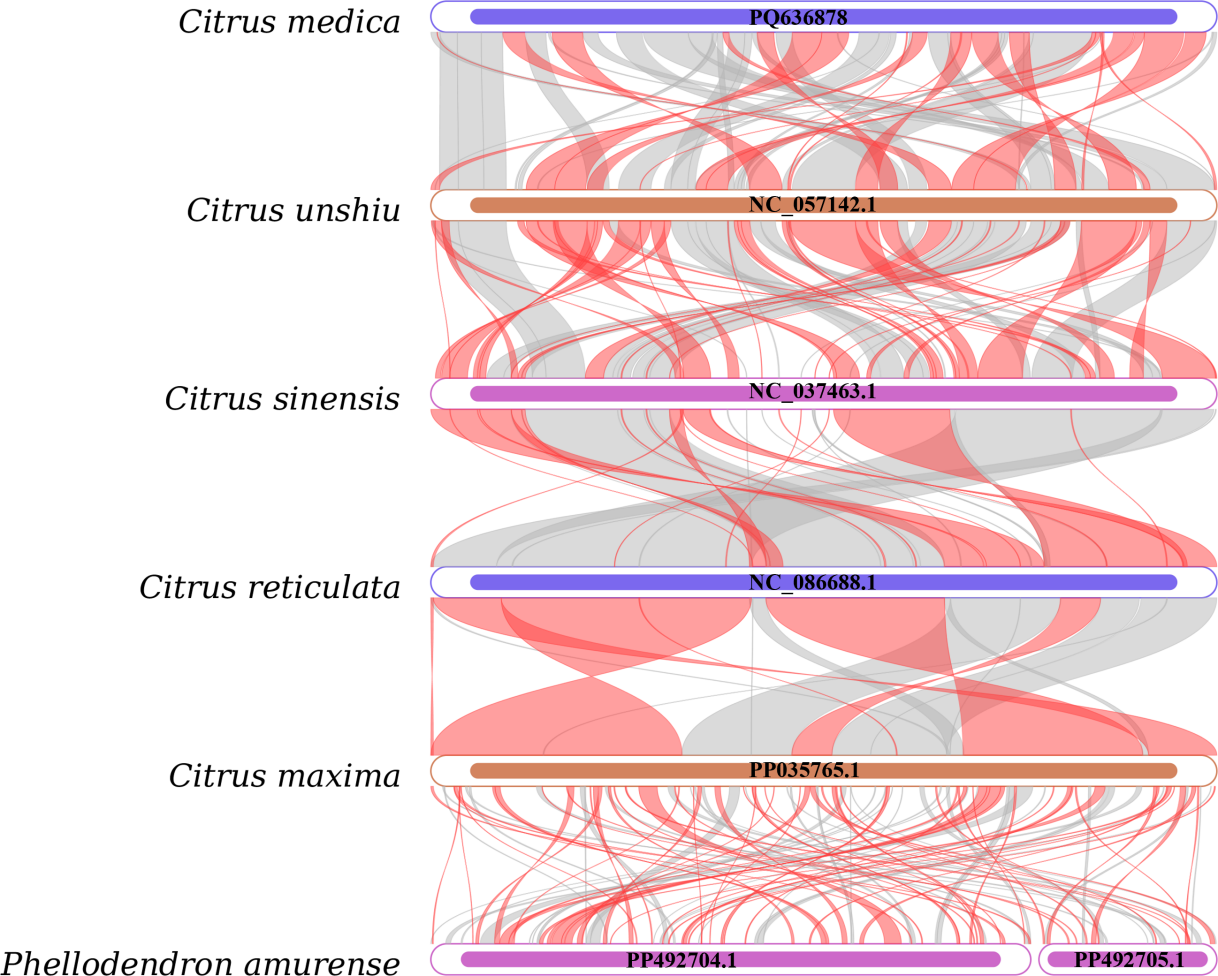
Collinear analysis of six Rutaceae species. Inverted regions (red arcs); homologous regions (gray arcs).

To assess sequence homology and structural conservation, a comparative analysis was conducted between the mitochondrial genomes of *C. medica* and five published Rutaceae species, employing BLASTN-based alignment of homologous genes and syntenic regions. Homologous sequences ≥300 bp in length were visualized (**Figure 5**), revealing abundant homologous fragments shared between *C. medica* and other Rutaceae species, with several sequences uniquely conserved within the *C. medica* mitochondrial genome. Furthermore, syntenic collinearity analysis detected significant structural divergence among the six mitochondrial genomes, characterized by multiple genomic rearrangement events coexisting with highly conserved regions.

### 3.4 Identification of RNA editing events

**Figure 6:**
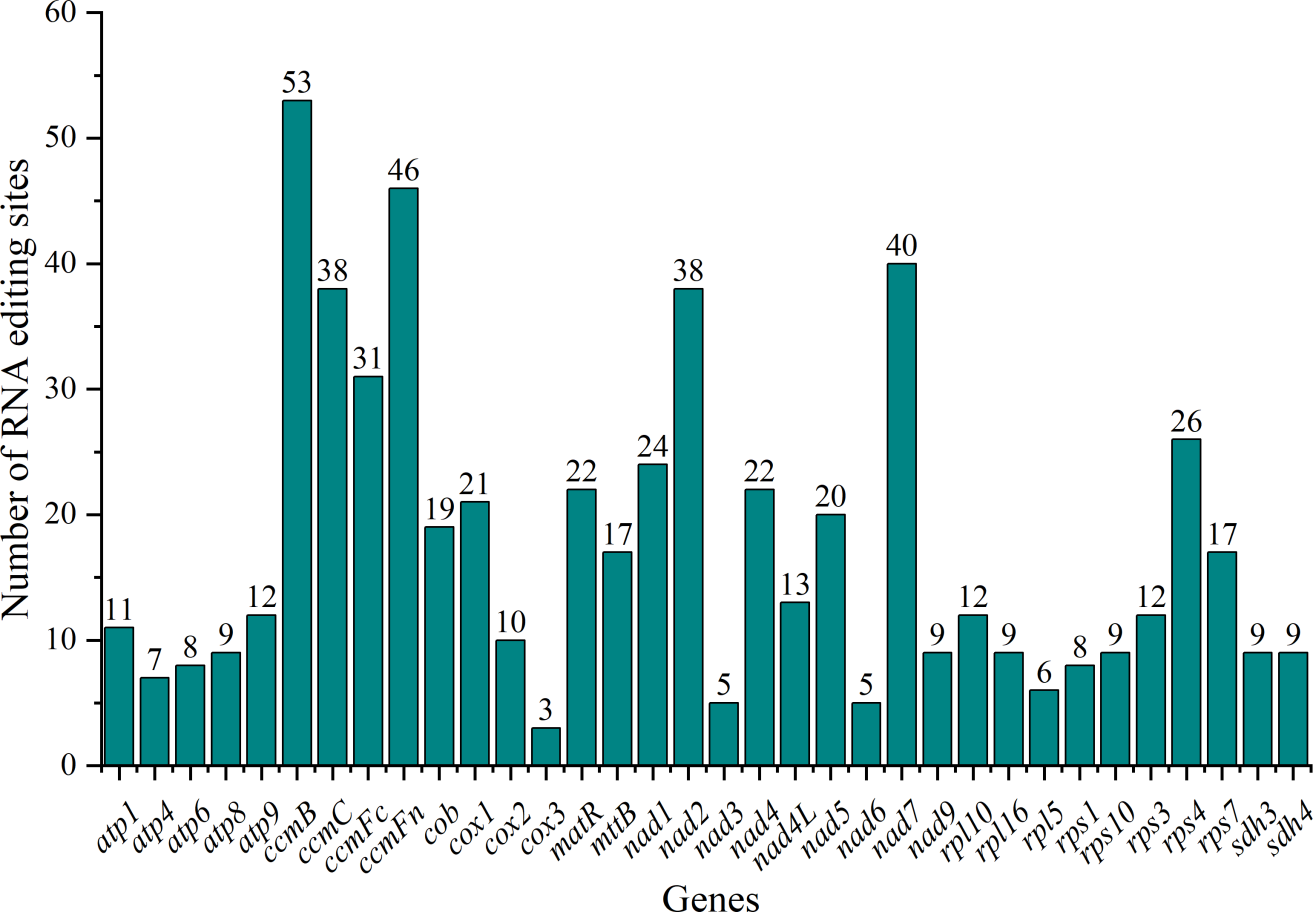
RNA editing sites in mitochondrial protein-coding genes (PCGs) of *C. medica.* The x-axis represents the name of the gene undergoing RNA editing. The y-axis represents the number of RNA editing sites.

We predicted RNA editing sites in 34 PCGs of the *C. medica* mitogenome to investigate genomic expression regulation. Our analysis identified 600 RNA editing events, predominantly characterized by C-to-T conversions. Among the 34 edited genes, ccmB exhibited the highest editing frequency with 53 identified sites, followed by ccmFn with 46 sites, whereas cox3 showed the lowest editing activity with only three modified sites (**Figure 6**). Positional analysis revealed distinct editing patterns: 198 events (33.00%) occurred at second codon positions, while third positions were more susceptible, with 266 events (44.33%). These modifications predominantly induced non-synonymous substitutions, including: histidine (H) to tyrosine (Y), arginine (R) to cysteine (C) or tryptophan (W), threonine (T) to isoleucine (I) or methionine (M), serine (S) to leucine (L) or phenylalanine (F), proline (P) to serine (S), leucine (L), or phenylalanine (F), leucine (L) to phenylalanine (F), alanine (A) to valine (V), premature stop codons from arginine (R) and glutamine (Q). Hydrophobicity analysis demonstrated that 33.00% of edits enhanced protein hydrophobicity (hydrophilic-to-hydrophobic transitions), potentially facilitating proper protein folding. Conversely, 5.83% introduced hydrophilic residues (hydrophobic-to-hydrophilic shifts), while 1.67% generated termination codons. The remaining 59.50% maintained original hydrophobicity profiles (**Supplementary Table S2**). Transcriptome analysis identified 114 RNA editing sites, of which 107 showed perfect concordance with Deepred-Mt predictions (**Supplementary Table S3**).

### 3.5 Analysis of codon usage bias and nucleotide polymorphism

**Figure 7:**
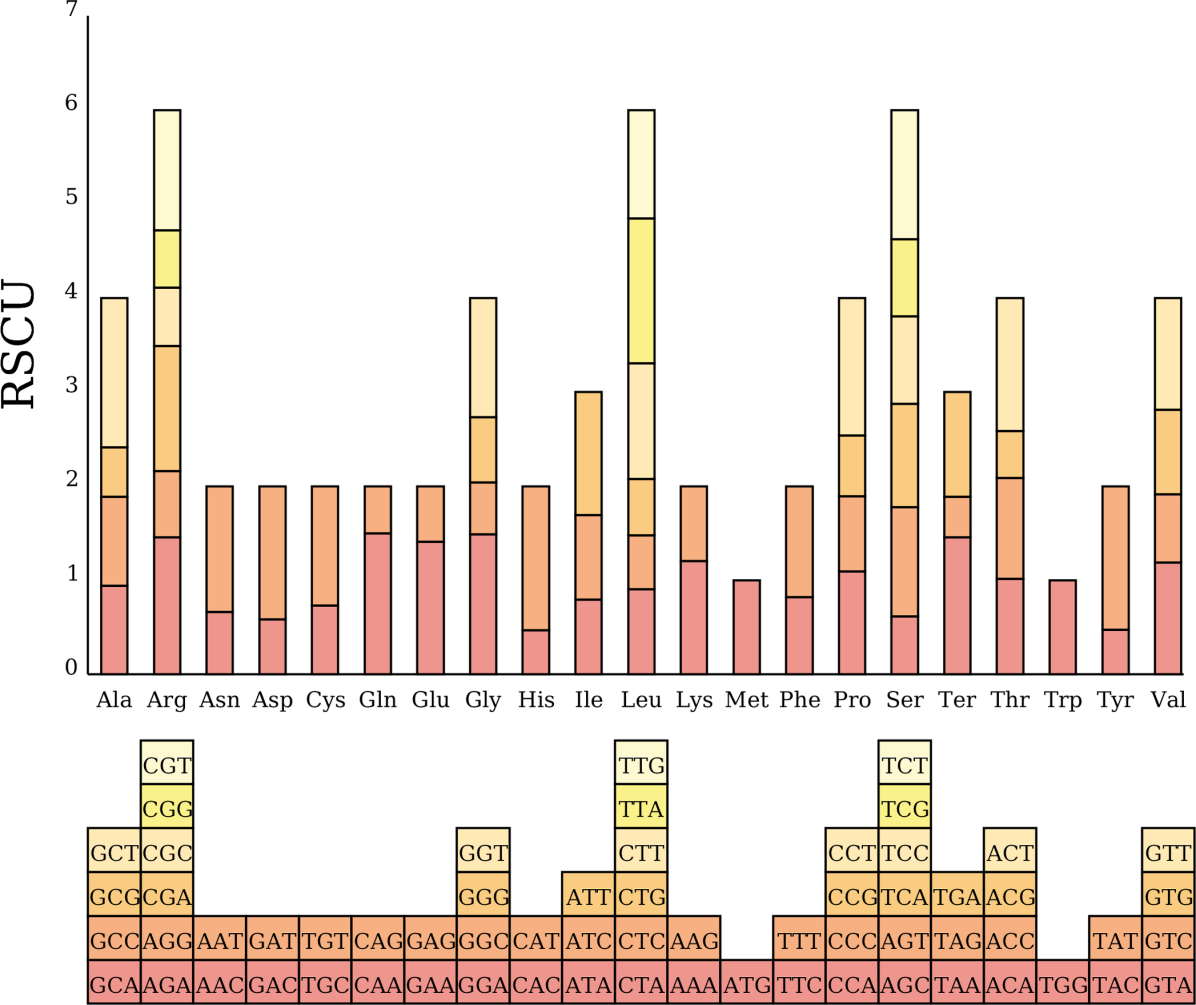
Codon usage bias in *C. medica* mitochondrial protein-coding genes (PCGs). RSCU values are represented on the y-axis, and the codons for the respective amino acids are represented on the x-axis.

**Figure 8:**
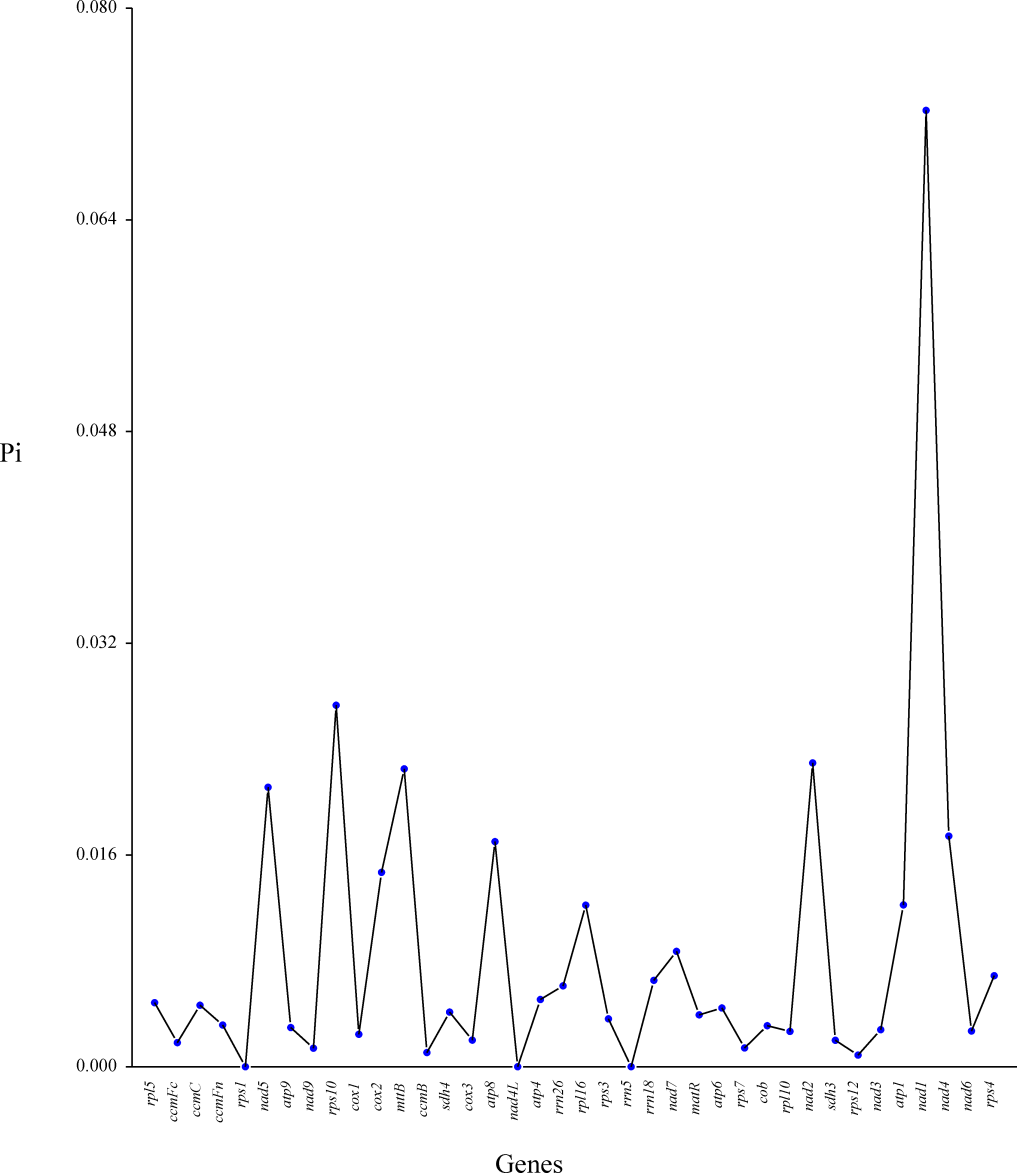
Nucleotide diversity (Pi) in the mitochondrial genome of *C. medica*. The x-axis represents mitochondrial genes, while the y-axis displays the corresponding Pi values for each gene.

We analyzed codon usage bias across 38 unique genes in the *C. medica* mitogenome, revealing a conserved preference pattern in protein-coding sequences. The start codon (AUG) and tryptophan codon (UGG) exhibited relative synonymous codon usage (RSCU) values of 1.0, while distinct biases were observed for other amino acids: alanine (Ala) showed the strongest preference for GCU (RSCU = 1.59), followed by leucine (Leu, RSCU = 1.54), and histidine (His) and tyrosine (Tyr) (both RSCU = 1.53). In contrast, phenylalanine (Phe) and valine (Val) exhibited weak codon biases, with maximum RSCU values below 1.2 (**Figure 7**; **Supplementary Table S4**). Additionally, nucleotide diversity (Pi) analysis of the mitogenome revealed values ranging from 0 to 0.07, with 15 protein-coding regions (*atp4*, *rrn26*, *rrn18*, *rps4*, *nad7*, r*pl16*, *atp1*, *cox2*, *atp8*, *nad4*, *nad5*, *mttB*, *nad2*, *rps10*, and *nad1*) exhibiting Pi values ≥ 0.01, indicative of elevated polymorphism levels (**Figure 8**; **Supplementary Table S5**).

### 3.6 Analysis of repetitive sequences in the mitochondrial genome of *C. medica*

**Figure 9:**
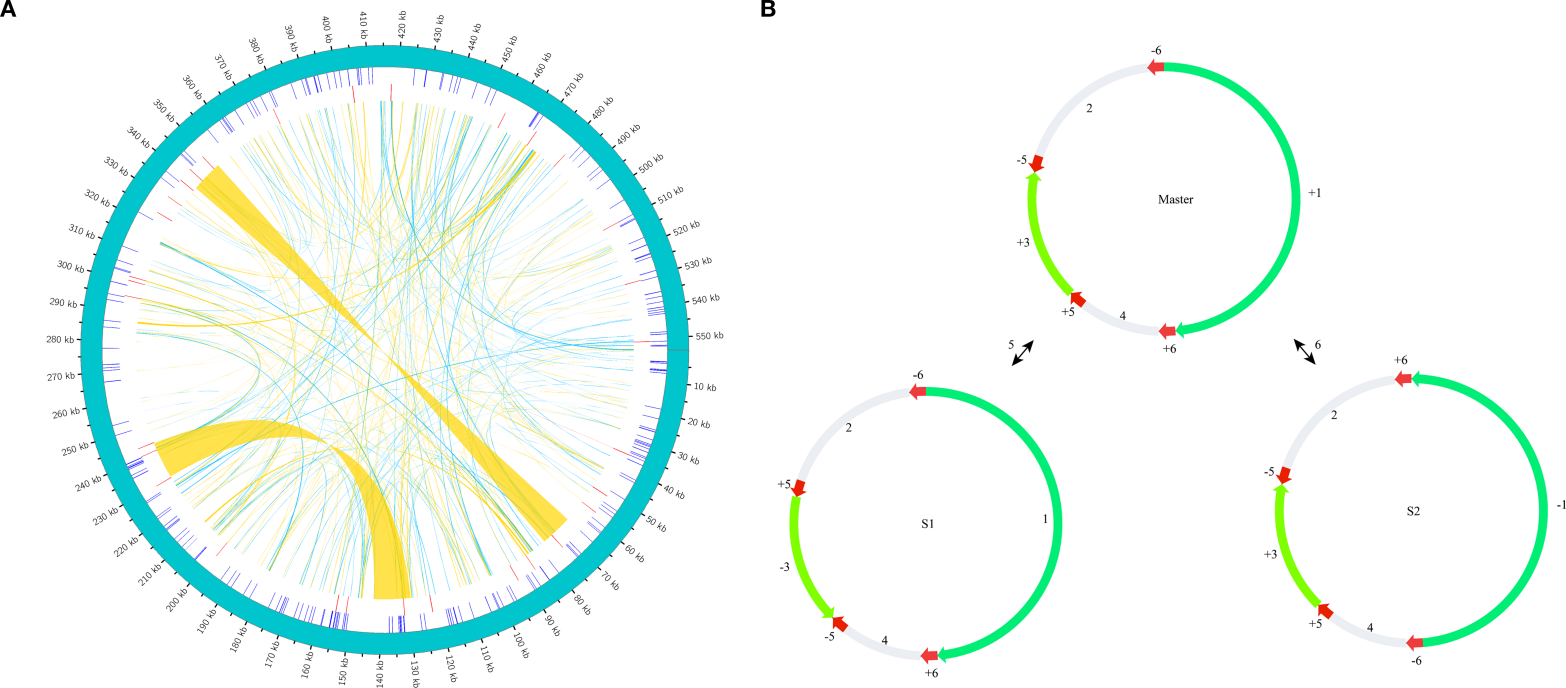
Repeat structures in the *C. medica* mitochondrial genome **(A)**. Schematic representation of repeat elements: the green outer arc depicts the complete mitochondrial genome, inner blue ticks indicate simple sequence repeats (SSRs), red ticks represent tandem repeats, and connecting ribbons show dispersed repeats **(A)**. Long direct repeat-mediated recombinational substructures, with red arrows marking the orientation and positions of repetitive sequences 5 and 6 involved in recombination events **(B)**.

**Figure 10:**
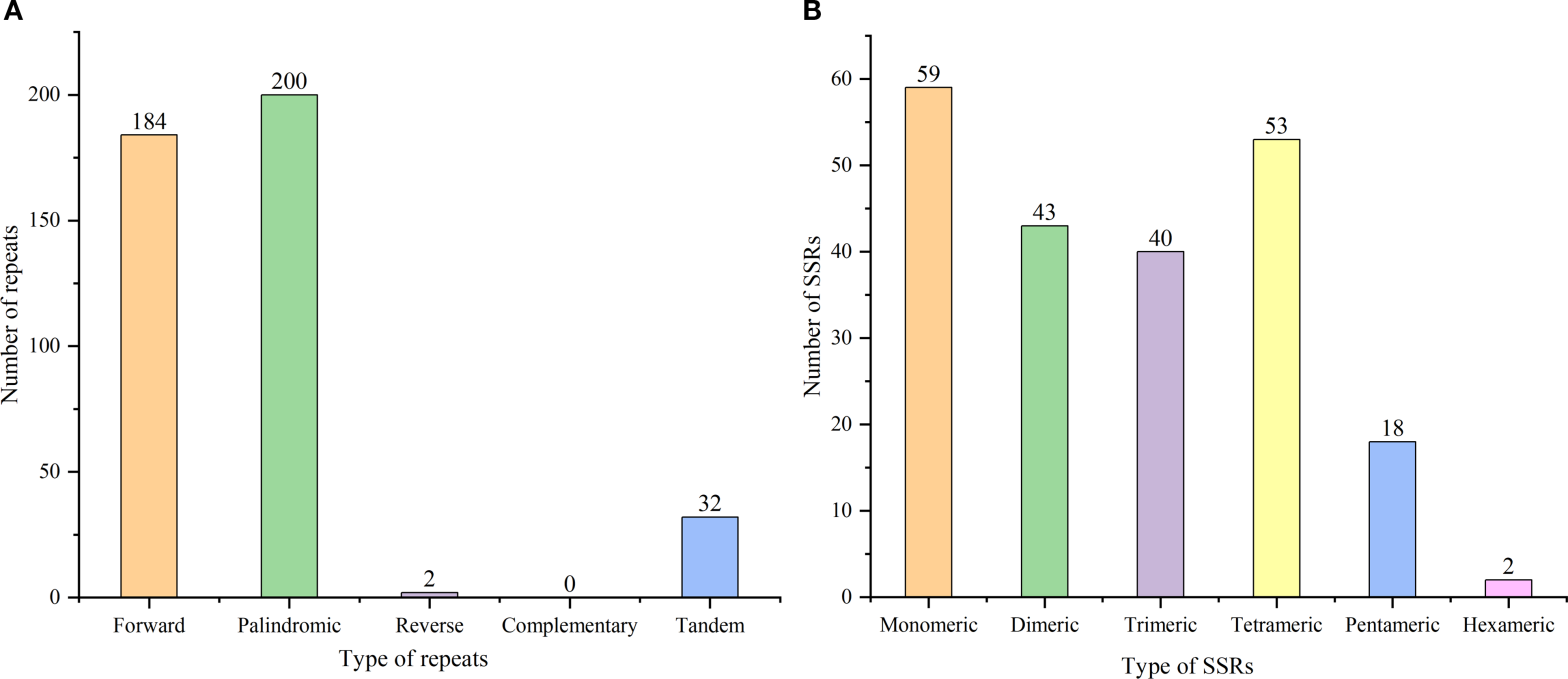
Repeat type distributions and abundances **(A)** and mitochondrial SSRs **(B)** in *C*. *medica*.

Our analysis identified 633 repetitive sequences within the *C. medica* mitochondrial genome, comprising 386 dispersed repeats, 215 SSRs, and 32 tandem repeats (**Figure 9A**). Notably, two long inverted complementary repeats (>1 kb) were detected (contigs 5 and 6 in **Supplementary Figure S1**), which may facilitate the formation of two distinct substructures (**Figure 9B**). Among the 386 dispersed repeats (>30 bp), 200 pairs were palindromic, 184 displayed forward orientation, and two were reverse, with none exhibiting complementary repeats (**Figure 10A**; **Supplementary Table S6**). The palindromic repeats included exceptionally long fragments (11,189 bp and 13,039 bp), while the longest forward repeat spanned 296 bp; reverse repeats measured 30–31 bp. Characterization of the 215 SSRs revealed the following distribution: 59 monomeric (27.44%), 43 dimeric (20.00%), 40 trimeric (18.60%), 53 tetrameric (24.65%), 18 pentameric (8.37%), and two hexameric (0.93%) (**Figure 10B**). Further analysis showed that 94.92% (56/59) of monomeric SSRs were A/T-rich, while 58.14% (25/43) of dimeric SSRs contained AG/CT motifs (**Supplementary Figure S5**). Additionally, 32 tandem repeats exhibited >71% sequence identity, with lengths ranging from 5 to 40 bp and copy numbers varying from 2 to 5 (**Supplementary Table S7**).

## 4 Discussion

The mitochondrial genome of *C. medica* assembled in this study represents the first complete mitochondrial genomic resource for this ancestral *Citrus* species. Our hybrid sequencing strategy, which integrates long-read Nanopore and short-read Illumina data, successfully reveals a mitochondrial genome size of 553,930 bp, consistent with the expansive genome architecture typical of plant mitochondria. Annotation of the genome revealed 65 functional genes, including 36 PCGs, 25 tRNAs, and 4 rRNAs. This gene repertoire aligns with the conserved patterns observed in other angiosperm mitochondrial genomes, ​​particularly the retention of 24 core PCGs (e.g., *atp1*, *cox1-3*, *nad1-9*) and convergent loss of ribosomal protein genes (*rps*/*rpl*) across land plants​ (Yu et al., 2018; Shen et al., 2022; Bi et al., 2023; Li et al., 2025).

The identification of 44 homologous fragments (totaling 41,479 bp) shared between the mitochondrial and chloroplast genomes provides compelling evidence for bidirectional DNA transfer. Notably, the presence of two exceptionally long homologous segments (6,767 bp each) transferred from the chloroplast genome highlights active inter-organellar DNA exchange. This phenomenon is increasingly recognized as a driver of mitochondrial genome plasticity in plants (Gandini et al., 2019; Odahara et al., 2024). Although these transferred fragments are non-functional within the mitochondrial genome, they may serve as reservoirs for evolutionary innovation through mechanisms such as exaptation or recombination-mediated structural variation (Gong et al., 2025). There exists a protein compensation mechanism between semi-autonomous organelles. For example, in *Arabidopsis thaliana*, *AtRNH1C* can substitute for the mitochondria-localized *AtRNH1B* to maintain genomic stability (Cheng et al., 2021; Duan et al., 2024). The lack of recent transfer events in *C. medica* contrasts with the frequent chloroplast-derived tRNA acquisitions noted in other *Citrus* species. This suggests *C. medica* may employ distinct mechanisms to maintain organellar genome stability, potentially linked to its unique evolutionary trajectory as a progenitor species (Huang et al., 2023).

The detection of 633 repetitive sequences, including long inverted repeats (>11 kb), provides mechanistic insights into the structural rearrangements and substoichiometric shifting observed in plant mitochondrial genomes. Such repeats may facilitate recombination events, generating subgenomic isoforms that contribute to genomic heteroplasmy—a critical feature for adaptive evolution in dynamic environments (Bentolila et al., 2002; Hao et al., 2024; Ma et al., 2025). The predominance of A/T-rich monomeric SSRs (94.92%) and AG/CT dimeric motifs aligns with the nucleotide bias typical of plant mitochondrial genomes, which may influence replication fidelity and genome stability.

The loss of the MutS Homolog 1 (MSH1) protein in *Arabidopsis thaliana* leads to an increase in abnormal recombination of medium-length repetitive sequences enriched in A/T nucleotides within the mitochondrial genome, coinciding with the accumulation of single-nucleotide variants and insertion/deletion mutations (indels). This finding underscores the sensitivity of A/T-rich regions in maintaining replication fidelity (Zou et al., 2022). Additionally, studies have shown that the accumulation of short repetitive sequences may be driven by slipped-strand mispairing, particularly those enriched in AG/CT dinucleotides. The structural properties of these sequences facilitate strand slippage during DNA replication, leading to the formation of localized repetitive elements that participate in the regulation of regional genomic stability (Alverson et al., 2010; Kong et al., 2025). These findings support the hypothesis that repetitive elements serve as hotspots for evolutionary innovation in organellar genomes.

RNA editing in plant mitochondria primarily occurs at the first and second positions of codons (>90%), resulting in changes to the physicochemical properties of amino acids (e.g., increased hydrophobicity) that are necessary for the structural stability of transmembrane proteins (Takenaka et al., 2008; Bi et al., 2023; Qin et al., 2024). Our RNA editing analysis identified 600 C-to-T conversion events, predominantly at the first and second codon positions, with significant impacts on amino acid hydrophobicity. The predominance of C-to-U conversions at second codon positions aligns with the RNA editing landscape of angiosperm mitochondria (Yang et al., 2017; Edera et al., 2021). The high editing frequency in *ccmB* (53 sites) and *ccmFn* (46 sites) suggests stringent post-transcriptional regulation of cytochrome c maturation genes, which are essential for maintaining electron transport chain efficiency (Faivre-Nitschke et al., 2001). Notably, 33% of these editing events increased amino acid hydrophobicity, potentially aiding in the proper folding of transmembrane proteins within oxidative phosphorylation complexes (Takenaka et al., 2008; Qin et al., 2024). These modifications are likely adaptive mechanisms that fine-tune protein function under fluctuating environmental conditions (Mower, 2009; Qin et al., 2024). Deepred-Mt demonstrates high predictive accuracy for RNA editing motifs, yet the quantitative discrepancy (600 predicted vs. 114 detected sites) primarily arises from methodological constraints. ​​We speculate​​ that oligo(dT)-based RNA-seq severely underrepresents non-polyadenylated mitochondrial transcripts, yielding insufficient coverage depth (~9×). This precludes reliable variant detection in regions below critical thresholds (≤5× coverage), masking genuine editing events despite genomic potential. If a whole-transcriptome sequencing strategy were employed—such as first removing rRNA followed by sequencing all RNAs without relying on poly(A) enrichment—it might be more ideal for validating RNA editing results. Furthermore, RNA editing is dynamically regulated by physiological or environmental factors—a dimension inherently unaddressed by static sequence-based predictions. These technical and biological limitations collectively explain the observed disparity.

Phylogenetic reconstruction firmly positions *C. medica* within the Rutaceae clade, demonstrating closer affinity to *C. sinensis* than to *C. maxima*. This topology reinforces *C. medica*’s status as a progenitor species of modern citrus hybrids, consistent with previous studies (Wu et al., 2018; Huang et al., 2023). However, the limited collinearity observed among the mitochondrial genomes of Rutaceae species highlights rapid structural divergence, likely driven by lineage-specific recombination and horizontal gene transfer events. Elevated nucleotide diversity (Pi ≥ 0.01) in 15 protein-coding regions, including *nad7* and *rps10*, highlights regions under relaxed selection, potentially serving as molecular markers for population-level studies.

## 5 Conclusion

This study establishes *C. medica* as a pivotal genomic resource for understanding organelle evolution in *Citrus*. The structural features of the mitochondrial genome, including chloroplast-derived sequences and recombination-prone repeats, provide a framework for investigating hybridization-driven diversification in commercially significant citrus hybrids. Moreover, the extensive RNA editing repertoire and codon usage bias patterns present insights into post-transcriptional regulatory mechanisms that may underlie adaptive traits. These findings not only address a critical gap in *Citrus* organelle genomics but also offer actionable data for molecular breeding programs aimed at enhancing stress tolerance and metabolic engineering in citrus crops.

## Data Availability

The original contributions presented in this study are publicly available. The assembled genome sequences have been deposited in NCBI GenBank (https://www.ncbi.nlm.nih.gov) under accession numbers PQ636878 and PP863286. Raw sequencing data are accessible under BioProject PRJNA1125280, with short-read data via BioSample SAMN41885986 (SRA accession: SRR29446786) and long-read data via BioSample SAMN49098229 (SRA accession: SRR33986094). The RNA-Seq data for C. medica supporting this study have been deposited in the NCBI Sequence Read Archive (SRA) under BioProject PRJNA1308020.
