# Supplementary material for: Assembly and comparative analysis of the first complete mitochondrial genome of Citrus medica (Rutaceae)
Source: Front Plant Sci. 2025 Sep 18;16:1649951. doi: 10.3389/fpls.2025.1649951 (PMC12488593; doi:10.3389/fpls.2025.1649951)
Supplement: Supplementary file 1 [file DataSheet1.docx]

Supplementary Material


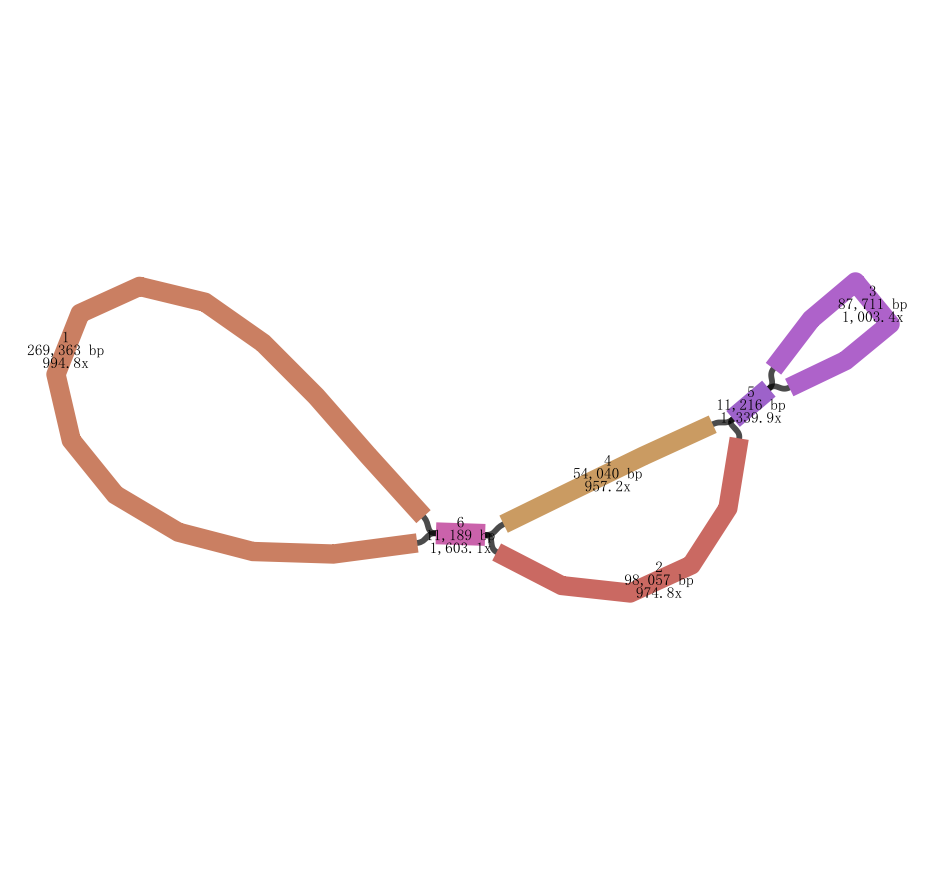


**Supplementary Figure 1**. Unitig graph of the *C. medica* mitochondrial genome. Contigs constituting the mitochondrial structure are represented by distinct colors and numerical labels, with corresponding lengths and average depths of coverage indicated. Contigs 5 and 6 form double-bifurcation structures.


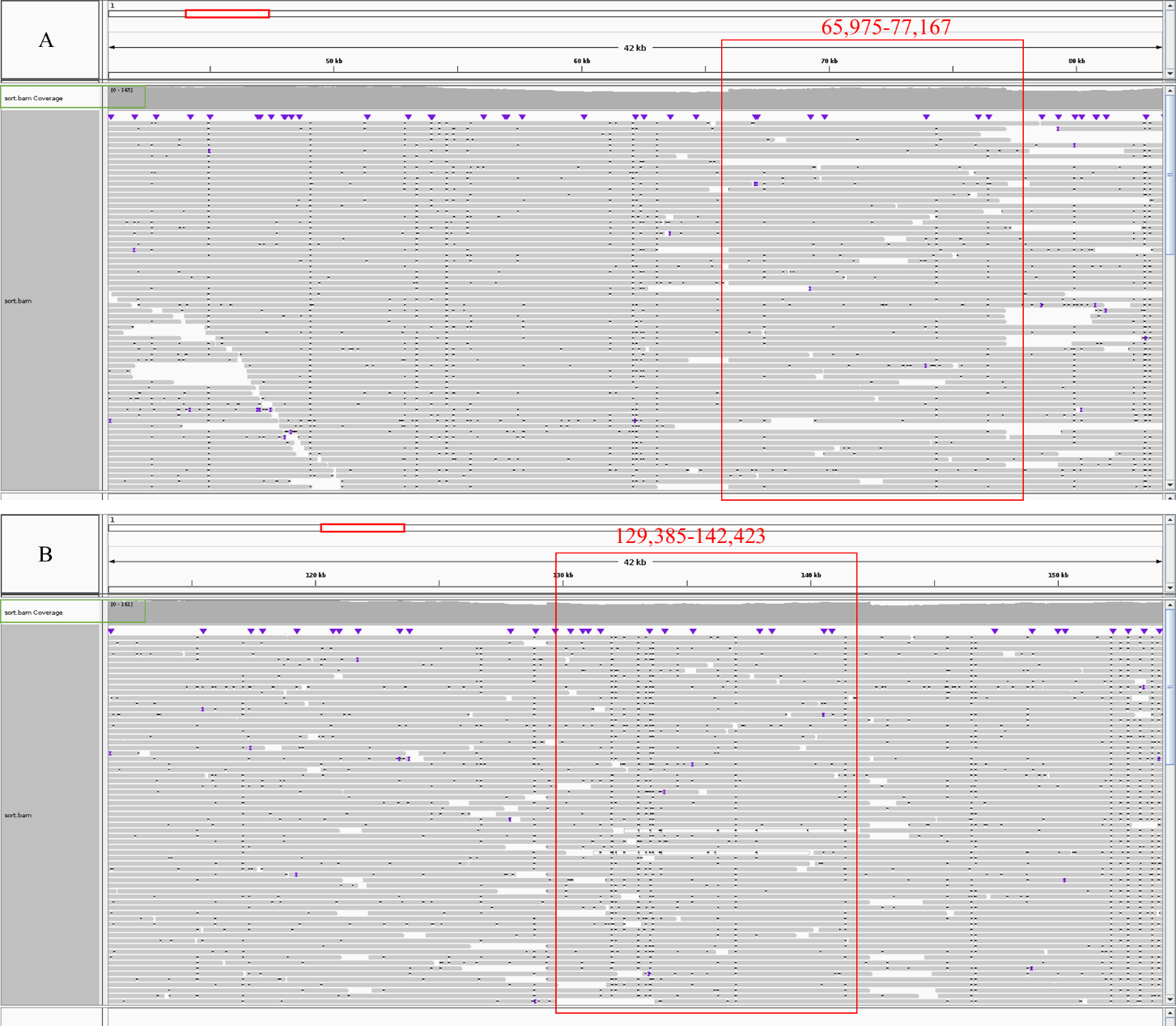


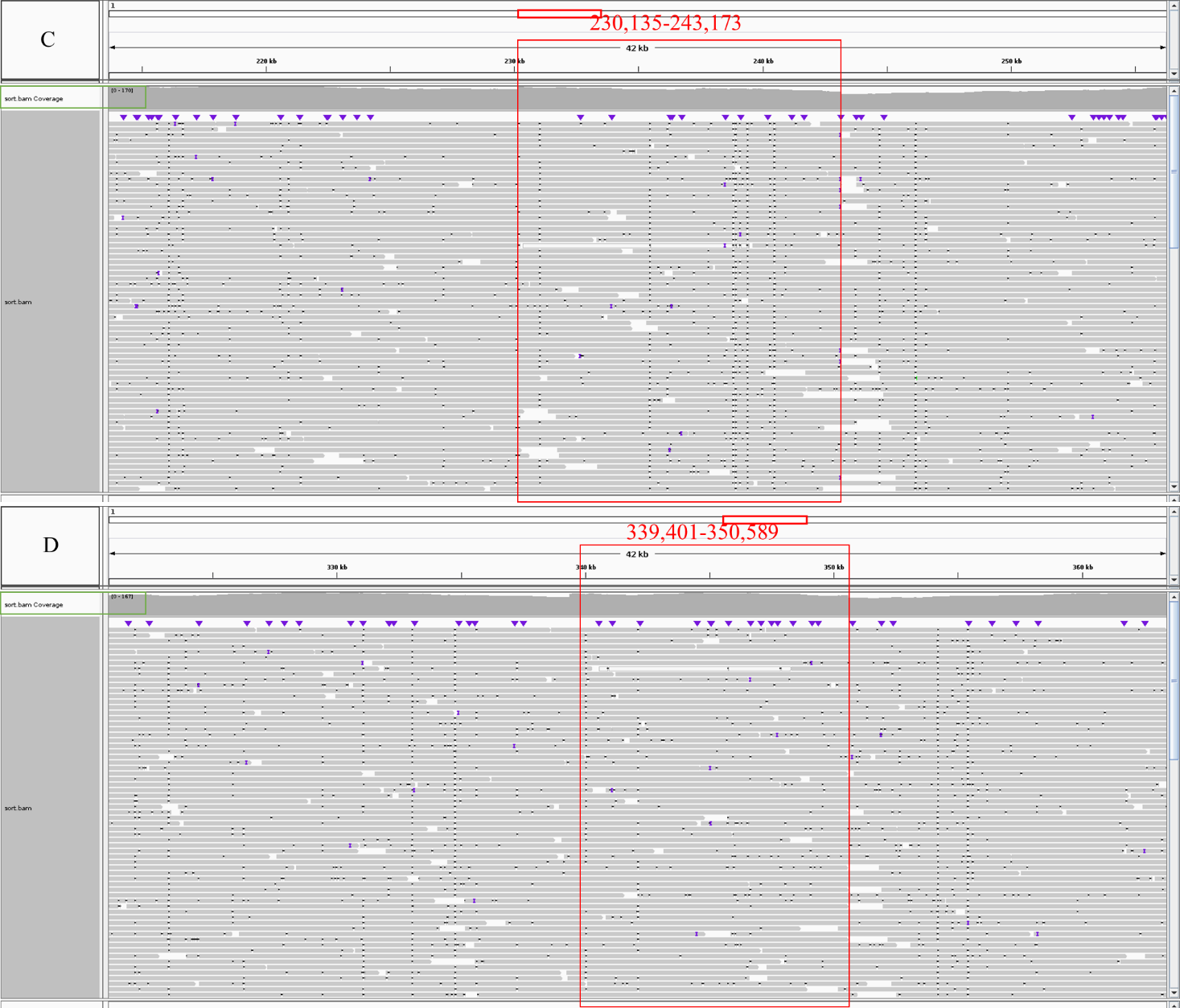


**Supplementary Figure 2.** IGV visualization of Nanopore reads spanning complete repeat junctions. Gray tracks show individual reads aligned across the repetitive region. Arrows in the gray tracks indicate the orientation of the reads. Red boxes and numbers in the figure represent the specific positions and ranges of repeat sequences in the genome, while green boxes and numbers indicate the range of read depth.


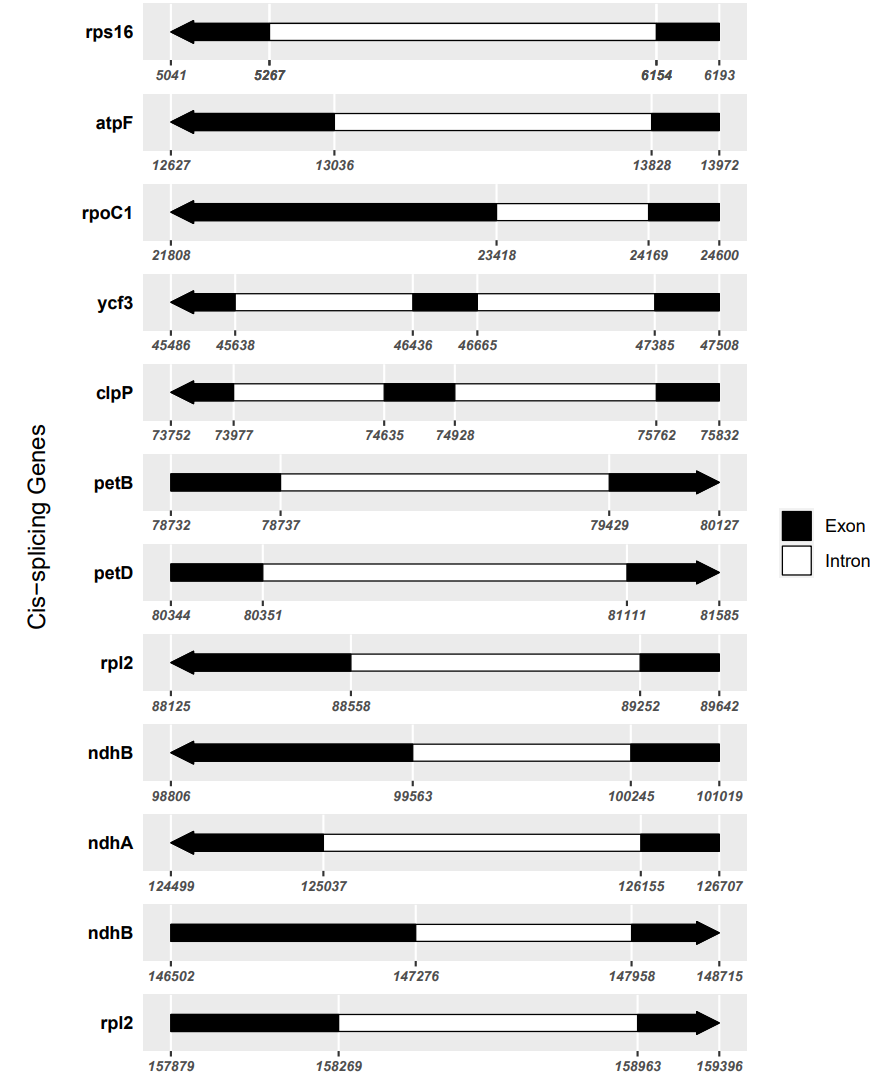


**Supplementary Figure 3.** The map of the cis-splicing genes in the *C. medica* chloroplast genome.


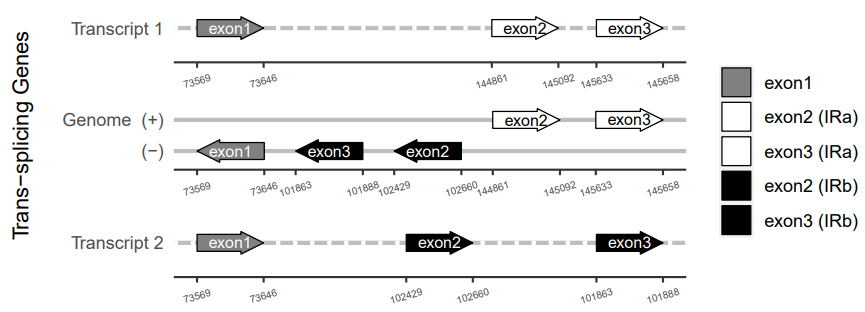


**Supplementary Figure 4.** The map of the trans-splicing gene *rps*12 in the *C. medica* chloroplast genome.


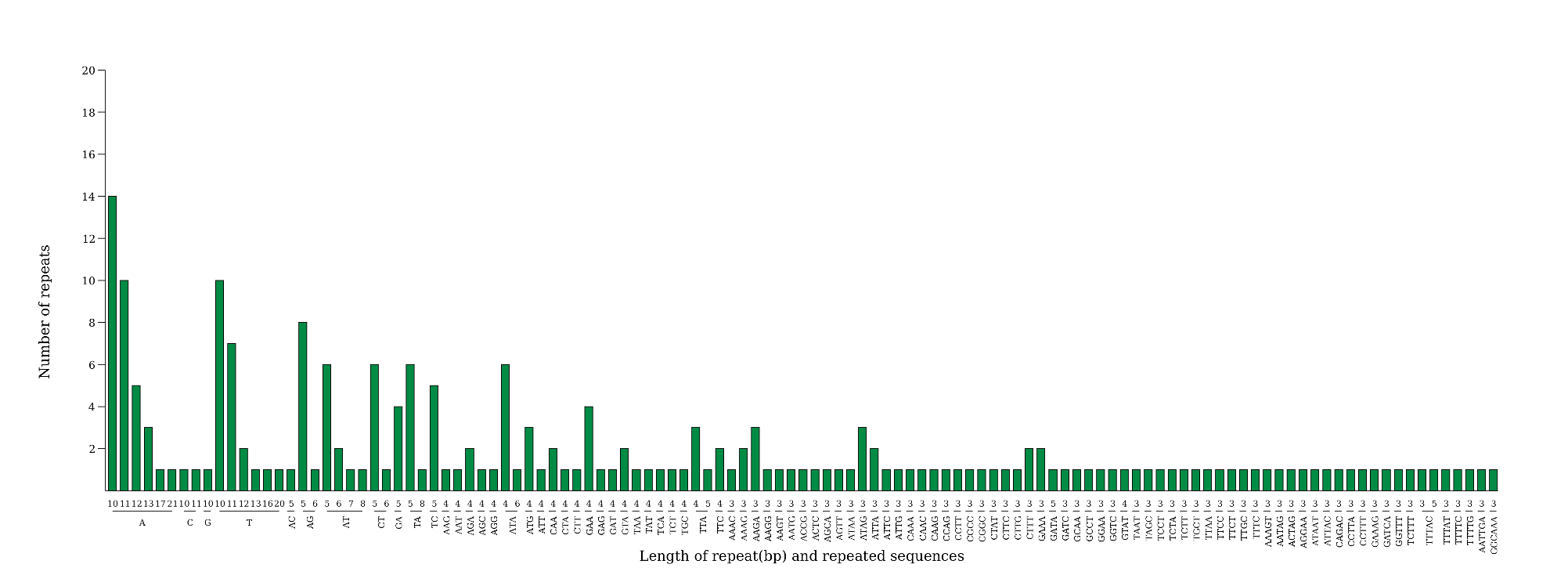


**Supplementary** **Figure 5.** The length and number of repeated sequences in the mitochondrial genome of *C. medica*.

**Supplementary Table 1. ​​**Comparative analysis of homology between the chloroplast and mitochondrial genomes of *C. medica*

| **Name** | **length** | **number of mismatches** | **start of alignment in query** | **end of alignment in query** | **start of alignment in subject** | **end of alignment in subject** | **gene(cp)** | **gene(mt)** |
| --- | --- | --- | --- | --- | --- | --- | --- | --- |
| CP1 | 6767 | 5 | 88572 | 95313 | 481308 | 474563 | rpl2(partical:70.55%);rpl23;trnI-CAU;ycf2(partical:73.65%) | trnM-CAT |
| CP2 | 6767 | 5 | 152208 | 158949 | 474563 | 481308 | ycf2(partical:73.65%);trnI-CAU;rpl23;rpl2(partical:70.55%) | trnM-CAT |
| CP3 | 2920 | 3 | 100208 | 103127 | 421969 | 424872 | ndhB(partical:36.68%);rps7;rps12(trans_splicing) | rps7 |
| CP4 | 2920 | 3 | 144394 | 147313 | 424872 | 421969 | rps12(trans_splicing);rps7;ndhB(partical:36.68%) | rps7 |
| CP5 | 2458 | 2 | 107055 | 109512 | 424861 | 427292 | trnI-GAU(partical:53.87%);trnA-UGC;rrn23(partical:28.52%) | trnA-TGC |
| CP6 | 2458 | 2 | 138009 | 140466 | 427292 | 424861 | rrn23(partical:28.52%);trnA-UGC;trnI-GAU(partical:53.87%) | trnA-TGC |
| CP7 | 2344 | 10 | 41490 | 43821 | 275589 | 273261 | psaB(partical:46.39%);psaA(partical:56.99%) |  |
| CP8 | 1262 | 34 | 59412 | 60653 | 333911 | 332668 | rbcL(partical:23.81%);accD(partical:23.53%) |  |
| CP9 | 1050 | 5 | 95281 | 96324 | 331235 | 332268 | ycf2(partical:15.24%) |  |
| CP10 | 1050 | 5 | 151197 | 152240 | 332268 | 331235 | ycf2(partical:15.24%) |  |
| CP11 | 937 | 5 | 109507 | 110443 | 448057 | 447121 | rrn23(partical:33.36%) |  |
| CP12 | 937 | 5 | 137078 | 138014 | 447121 | 448057 | rrn23(partical:33.36%) |  |
| CP13 | 918 | 16 | 40543 | 41454 | 276500 | 275583 | psaB(partical:41.36%) |  |
| CP14 | 879 | 171 | 105006 | 105859 | 374245 | 373397 | rrn16(partical:57.28%) | rrn18(partical:43.58%) |
| CP15 | 879 | 171 | 141662 | 142515 | 373397 | 374245 | rrn16(partical:57.28%) | rrn18(partical:43.58%) |
| CP16 | 806 | 4 | 103793 | 104582 | 336069 | 335270 | trnV-GAC | trnV-GAC |
| CP17 | 806 | 4 | 142939 | 143728 | 335270 | 336069 | trnV-GAC | trnV-GAC |
| CP18 | 760 | 19 | 26553 | 27312 | 323927 | 323169 | rpoB(partical:23.65%) |  |
| CP19 | 515 | 41 | 47095 | 47601 | 165433 | 165932 | ycf3(partical:20.46%) |  |
| CP20 | 492 | 14 | 58613 | 59102 | 359443 | 359901 | rbcL(partical:34.31%) |  |
| CP21 | 423 | 7 | 60932 | 61354 | 332665 | 332262 | accD(partical:28.60%) |  |
| CP22 | 390 | 50 | 70093 | 70478 | 108711 | 108348 | trnW-CCA;trnP-UGG | trnP-TGG;trnW-CCA |
| CP23 | 299 | 8 | 99667 | 99959 | 130199 | 129909 | ndhB(partical:13.23%) |  |
| CP24 | 299 | 8 | 99667 | 99959 | 242359 | 242649 | ndhB(partical:13.23%) |  |
| CP25 | 299 | 8 | 147562 | 147854 | 129909 | 130199 | ndhB(partical:13.23%) |  |
| CP26 | 299 | 8 | 147562 | 147854 | 242649 | 242359 | ndhB(partical:13.23%) |  |
| CP27 | 188 | 13 | 47864 | 48044 | 166630 | 166817 | trnS-GGA | trnS-GGA |
| CP28 | 97 | 16 | 109133 | 109229 | 72458 | 72554 | rrn23(partical:3.45%) | rrn26(partical:3.10%) |
| CP29 | 97 | 16 | 109133 | 109229 | 344110 | 344014 | rrn23(partical:3.45%) | rrn26(partical:3.10%) |
| CP30 | 97 | 16 | 138292 | 138388 | 72554 | 72458 | rrn23(partical:3.45%) | rrn26(partical:3.10%) |
| CP31 | 97 | 16 | 138292 | 138388 | 344014 | 344110 | rrn23(partical:3.45%) | rrn26(partical:3.10%) |
| CP32 | 91 | 16 | 8804 | 8893 | 166776 | 166688 | trnS-GCU | trnS-GGA |
| CP33 | 87 | 4 | 90107 | 90181 | 46003 | 45917 | trnI-CAU | ccmC(partical:1.86%);trnI-TAT |
| CP34 | 87 | 4 | 157340 | 157414 | 45917 | 46003 | trnI-CAU | ccmC(partical:1.86%);trnI-TAT |
| CP35 | 84 | 2 | 112973 | 113055 | 515214 | 515131 | trnN-GUU | trnN-GTT |
| CP36 | 84 | 2 | 134466 | 134548 | 515131 | 515214 | trnN-GUU | trnN-GTT |
| CP37 | 81 | 3 | 16 | 96 | 156989 | 156909 | trnH-GUG | trnH-GTG |
| CP38 | 81 | 2 | 32531 | 32611 | 534076 | 534156 | trnD-GUC | trnD-GTC |
| CP39 | 79 | 4 | 55326 | 55404 | 162402 | 162479 | trnM-CAU | trnM-CAT |
| CP40 | 78 | 4 | 95236 | 95313 | 376891 | 376968 | ycf2(partical:1.14%) |  |
| CP41 | 78 | 4 | 152208 | 152285 | 376968 | 376891 | ycf2(partical:1.14%) |  |
| CP42 | 58 | 3 | 11226 | 11283 | 307760 | 307817 | atpA(partical:3.81%) |  |
| CP43 | 41 | 1 | 125076 | 125116 | 424450 | 424490 | ndhA(partical:1.86%) |  |
| CP44 | 40 | 0 | 26527 | 26566 | 323923 | 323962 | rpoB(partical:1.24%) |  |

**Supplementary Table 2.** RNA editing events predicted in *C. medica* mitochondrial genome using Deepred-Mt website

| **Type** | **RNA-editing** | **Number** | **Percentage** |
| --- | --- | --- | --- |
| hydrophilic-hydrophilic | AAC (N) => AAT (N) | 4 |  |
|  | ACC (T) => ACT (T) | 10 |  |
|  | CAC (H) => TAC (Y) | 5 |  |
|  | CAT (H) => TAT (Y) | 13 |  |
|  | CGC (R) => TGC (C) | 8 |  |
|  | CGT (R) => TGT (C) | 26 |  |
|  | GAC (D) => GAT (D) | 2 |  |
|  | TAC (Y) => TAT (Y) | 5 |  |
|  | TCC (S) => TCT (S) | 17 |  |
|  | TGC (C) => TGT (C) | 1 |  |
|  | total | 91 | 15.17% |
| hydrophilic-hydrophobic | ACA (T) => ATA (I) | 4 |  |
|  | ACC (T) => ATC (I) | 3 |  |
|  | ACG (T) => ATG (M) | 2 |  |
|  | ACT (T) => ATT (I) | 6 |  |
|  | CGG (R) => TGG (W) | 24 |  |
|  | TCA (S) => TTA (L) | 52 |  |
|  | TCC (S) => TTC (F) | 18 |  |
|  | TCC (S) => TTT (F) | 18 |  |
|  | TCG (S) => TTG (L) | 38 |  |
|  | TCT (S) => TTT (F) | 33 |  |
|  | total | 198 | 33.00% |
| hydrophilic-stop | CAA (Q) => TAA (X) | 3 |  |
|  | CAG (Q) => TAG (X) | 2 |  |
|  | CGA (R) => TGA (X) | 5 |  |
|  | total | 10 | 1.67% |
| hydrophobic-hydrophilic | CCA (P) => TCA (S) | 5 |  |
|  | CCC (P) => TCC (S) | 6 |  |
|  | CCC (P) => TCT (S) | 2 |  |
|  | CCG (P) => TCG (S) | 5 |  |
|  | CCT (P) => TCT (S) | 17 |  |
|  | total | 35 | 5.83% |
| hydrophobic-hydrophobic | ATC (I) => ATT (I) | 30 |  |
|  | CCA (P) => CTA (L) | 23 |  |
|  | CCA (P) => TTA (L) | 20 |  |
|  | CCC (P) => CCT (P) | 11 |  |
|  | CCC (P) => CTC (L) | 5 |  |
|  | CCC (P) => CTT (L) | 2 |  |
|  | CCC (P) => TTC (F) | 4 |  |
|  | CCG (P) => CTG (L) | 14 |  |
|  | CCG (P) => TTG (L) | 10 |  |
|  | CCT (P) => CTT (L) | 14 |  |
|  | CCT (P) => TTT (F) | 8 |  |
|  | CTA (L) => TTA (L) | 12 |  |
|  | CTC (L) => CTT (L) | 16 |  |
|  | CTC (L) => TTC (F) | 5 |  |
|  | CTC (L) => TTT (F) | 2 |  |
|  | CTG (L) => TTG (L) | 13 |  |
|  | CTT (L) => TTT (F) | 13 |  |
|  | GCA (A) => GTA (V) | 2 |  |
|  | GCC (A) => GCT (A) | 5 |  |
|  | GCC (A) => GTC (V) | 2 |  |
|  | GCG (A) => GTG (V) | 4 |  |
|  | GCT (A) => GTT (V) | 1 |  |
|  | GGC (G) => GGT (G) | 2 |  |
|  | GTC (V) => GTT (V) | 6 |  |
|  | TTC (F) => TTT (F) | 42 |  |
|  | total | 266 | 44.33% |

**Supplementary Table 3.** Validation of RNA editing events in *C. medica* mitochondrial genome by RNA-seq mapping​ ​

| Type | RNA-editing | Number | Percentage |
| --- | --- | --- | --- |
| hydrophilic-hydrophilic | CAC(H) => TAC(Y) | 4 |  |
|  | CAT(H) => TAT(Y) | 5 |  |
|  | CGT(R) => TGT(C) | 5 |  |
|  | TCC(S) => TCT(S) | 2 |  |
|  | TCG(S) => TCC(S) | 1 |  |
|  | TCG(S) => TCT(S) | 1 |  |
|  | total | 18 | 15.79% |
| hydrophilic-hydrophobic | ACA(T) => ATA(I) | 1 |  |
|  | CGG(R) => TGG(W) | 11 |  |
|  | TCA(S) => TTA(L) | 17 |  |
|  | TCC(S) => TTC(F) | 5 |  |
|  | TCG(S) => TTG(L) | 8 |  |
|  | TCT(S) => TTT(F) | 12 |  |
|  | total | 54 | 47.37% |
| hydrophilic-stop | CGA(R) => TGA(*) | 1 |  |
|  | total | 1 | 0.88% |
| hydrophobic-hydrophilic | CCA(P) => TCA(S) | 2 |  |
|  | CCC(P) => TCC(S) | 2 |  |
|  | CCG(P) => TCG(S) | 1 |  |
|  | CCT(P) => TCT(S) | 6 |  |
|  | total | 11 | 9.65% |
| hydrophobic-hydrophobic | ATC(I) => ATT(I) | 1 |  |
|  | CCA(P) => CTA(L) | 10 |  |
|  | CCC(P) => CTC(L) | 1 |  |
|  | CCC(P) => CTT(L) | 2 |  |
|  | CCG(P) => CTG(L) | 4 |  |
|  | CCT(P) => CTT(L) | 1 |  |
|  | CTA(L) => TTA(L) | 1 |  |
|  | CTC(L) => CTT(L) | 2 |  |
|  | CTC(L) => TTC(F) | 2 |  |
|  | CTG(L) => TTG(L) | 1 |  |
|  | CTT(L) => GTT(V) | 1 |  |
|  | CTT(L) => TTT(F) | 1 |  |
|  | GCG(A) => GTG(V) | 1 |  |
|  | TTC(F) => TTT(F) | 2 |  |
|  | total | 30 | 26.32% |

**Supplementary Table 4.** Codon preference in protein-coding genes of the *C. medica* mitochondrial genome

| **AminoAcid** | **Symbol** | **Codon** | **No.** | **RSCU** |
| --- | --- | --- | --- | --- |
| * | Ter | UAA | 17 | 1.46 |
| * | Ter | UAG | 5 | 0.43 |
| * | Ter | UGA | 13 | 1.11 |
| A | Ala | GCA | 150 | 0.94 |
| A | Ala | GCC | 151 | 0.95 |
| A | Ala | GCG | 84 | 0.53 |
| A | Ala | GCU | 253 | 1.59 |
| C | Cys | UGC | 56 | 0.73 |
| C | Cys | UGU | 97 | 1.27 |
| D | Asp | GAC | 93 | 0.59 |
| D | Asp | GAU | 224 | 1.41 |
| E | Glu | GAA | 305 | 1.41 |
| E | Glu | GAG | 127 | 0.59 |
| F | Phe | UUC | 288 | 0.82 |
| F | Phe | UUU | 412 | 1.18 |
| G | Gly | GGA | 261 | 1.49 |
| G | Gly | GGC | 97 | 0.55 |
| G | Gly | GGG | 122 | 0.70 |
| G | Gly | GGU | 222 | 1.27 |
| H | His | CAC | 57 | 0.47 |
| H | His | CAU | 187 | 1.53 |
| I | Ile | AUA | 210 | 0.80 |
| I | Ile | AUC | 236 | 0.90 |
| I | Ile | AUU | 344 | 1.31 |
| K | Lys | AAA | 264 | 1.21 |
| K | Lys | AAG | 174 | 0.79 |
| L | Leu | CUA | 177 | 0.91 |
| L | Leu | CUC | 112 | 0.57 |
| L | Leu | CUG | 117 | 0.60 |
| L | Leu | CUU | 241 | 1.23 |
| L | Leu | UUA | 301 | 1.54 |
| L | Leu | UUG | 225 | 1.15 |
| M | Met | AUG | 275 | 1.00 |
| N | Asn | AAC | 111 | 0.66 |
| N | Asn | AAU | 224 | 1.34 |
| P | Pro | CCA | 139 | 1.09 |
| P | Pro | CCC | 102 | 0.80 |
| P | Pro | CCG | 82 | 0.64 |
| P | Pro | CCU | 186 | 1.46 |
| Q | Gln | CAA | 217 | 1.50 |
| Q | Gln | CAG | 72 | 0.50 |
| R | Arg | AGA | 160 | 1.46 |
| R | Arg | AGG | 77 | 0.70 |
| R | Arg | CGA | 146 | 1.33 |
| R | Arg | CGC | 68 | 0.62 |
| R | Arg | CGG | 67 | 0.61 |
| R | Arg | CGU | 140 | 1.28 |
| S | Ser | AGC | 89 | 0.62 |
| S | Ser | AGU | 168 | 1.16 |
| S | Ser | UCA | 159 | 1.10 |
| S | Ser | UCC | 135 | 0.93 |
| S | Ser | UCG | 118 | 0.82 |
| S | Ser | UCU | 199 | 1.38 |
| T | Thr | ACA | 132 | 1.02 |
| T | Thr | ACC | 139 | 1.07 |
| T | Thr | ACG | 65 | 0.50 |
| T | Thr | ACU | 183 | 1.41 |
| V | Val | GUA | 190 | 1.19 |
| V | Val | GUC | 116 | 0.73 |
| V | Val | GUG | 143 | 0.90 |
| V | Val | GUU | 190 | 1.19 |
| W | Trp | UGG | 159 | 1.00 |
| Y | Tyr | UAC | 77 | 0.47 |
| Y | Tyr | UAU | 248 | 1.53 |

**Supplementary Table 5.** Nucleotide diversity (Pi) analysis in the mitochondrial genome of *C. medica​​*

| **No.** | **Region** | **Pi** | **Total Number of mutations** | **Region length** |
| --- | --- | --- | --- | --- |
| 1 | gene1.rpl5 | 0.00 | 6 | 558 |
| 2 | gene10.cox1 | 0.00 | 9 | 1653 |
| 3 | gene11.cox2 | 0.01 | 28 | 798 |
| 4 | gene12.mttB | 0.02 | 15 | 982 |
| 5 | gene13.ccmB | 0.00 | 2 | 621 |
| 6 | gene14.sdh4 | 0.00 | 4 | 618 |
| 7 | gene15.cox3 | 0.00 | 4 | 798 |
| 8 | gene16.atp8 | 0.02 | 16 | 492 |
| 9 | gene17.nad4L | 0.00 | 0 | 303 |
| 10 | gene18.atp4 | 0.01 | 9 | 597 |
| 11 | gene19.rrn26 | 0.01 | 30 | 3397 |
| 12 | gene2.ccmFc | 0.00 | 6 | 1476 |
| 13 | gene20.rpl16 | 0.01 | 15 | 558 |
| 14 | gene21.rps3 | 0.00 | 11 | 1683 |
| 15 | gene22.rrn5 | 0.00 | 0 | 119 |
| 16 | gene23.rrn18 | 0.01 | 36 | 1953 |
| 17 | gene24.nad7 | 0.01 | 18 | 1186 |
| 18 | gene25.matR | 0.00 | 15 | 2802 |
| 19 | gene26.atp6 | 0.00 | 8 | 849 |
| 20 | gene27.rps7 | 0.00 | 1 | 468 |
| 21 | gene28.cob | 0.00 | 7 | 1182 |
| 22 | gene29.rpl10 | 0.00 | 3 | 489 |
| 23 | gene3.ccmC | 0.00 | 7 | 753 |
| 24 | gene30.nad2 | 0.02 | 36 | 1467 |
| 25 | gene31.sdh3 | 0.00 | 1 | 333 |
| 26 | gene32.rps12 | 0.00 | 1 | 378 |
| 27 | gene33.nad3 | 0.00 | 3 | 357 |
| 28 | gene34.atp1 | 0.01 | 48 | 1536 |
| 29 | gene35.nad1 | 0.07 | 94 | 999 |
| 30 | gene36.nad4 | 0.02 | 33 | 1609 |
| 31 | gene37.nad6 | 0.00 | 5 | 618 |
| 32 | gene38.rps4 | 0.01 | 18 | 1053 |
| 33 | gene4.ccmFn | 0.00 | 13 | 1751 |
| 34 | gene5.rps1 | 0.00 | 0 | 615 |
| 35 | gene6.nad5 | 0.02 | 75 | 2016 |
| 36 | gene7.atp9 | 0.00 | 2 | 291 |
| 37 | gene8.nad9 | 0.00 | 2 | 573 |
| 38 | gene9.rps10 | 0.03 | 15 | 450 |

**Supplementary Table 6.** Characterization of repetitive elements in the mitochondrial genome of *C. medica​*

| **Length (bp)** | **Forward repeat** | **Palindromic repeat** | **Reverse repeat** | **Complementary repeat** | **Total** |
| --- | --- | --- | --- | --- | --- |
| 30 | 39 | 39 | 1 | 0 | 79 |
| 31 | 25 | 16 | 1 | 0 | 42 |
| 32 | 13 | 14 | 0 | 0 | 27 |
| 33 | 10 | 17 | 0 | 0 | 27 |
| 34 | 5 | 11 | 0 | 0 | 16 |
| 35 | 8 | 7 | 0 | 0 | 15 |
| 36 | 4 | 5 | 0 | 0 | 9 |
| 37 | 7 | 3 | 0 | 0 | 10 |
| 38 | 7 | 3 | 0 | 0 | 10 |
| 39 | 1 | 3 | 0 | 0 | 4 |
| 40 | 3 | 3 | 0 | 0 | 6 |
| 41 | 6 | 1 | 0 | 0 | 7 |
| 42 | 2 | 4 | 0 | 0 | 6 |
| 43 | 1 | 4 | 0 | 0 | 5 |
| 44 | 1 | 1 | 0 | 0 | 2 |
| 45 | 3 | 2 | 0 | 0 | 5 |
| 46 | 3 | 4 | 0 | 0 | 7 |
| 47 | 0 | 2 | 0 | 0 | 2 |
| 48 | 3 | 1 | 0 | 0 | 4 |
| 49 | 3 | 1 | 0 | 0 | 4 |
| 50 | 0 | 3 | 0 | 0 | 3 |
| 52 | 3 | 1 | 0 | 0 | 4 |
| 53 | 6 | 1 | 0 | 0 | 7 |
| 55 | 0 | 2 | 0 | 0 | 2 |
| 56 | 1 | 1 | 0 | 0 | 2 |
| 57 | 2 | 2 | 0 | 0 | 4 |
| 58 | 1 | 1 | 0 | 0 | 2 |
| 59 | 2 | 1 | 0 | 0 | 3 |
| 62 | 1 | 1 | 0 | 0 | 2 |
| 63 | 1 | 0 | 0 | 0 | 1 |
| 64 | 0 | 2 | 0 | 0 | 2 |
| 65 | 0 | 1 | 0 | 0 | 1 |
| 67 | 1 | 0 | 0 | 0 | 1 |
| 70 | 2 | 0 | 0 | 0 | 2 |
| 71 | 0 | 1 | 0 | 0 | 1 |
| 73 | 0 | 1 | 0 | 0 | 1 |
| 74 | 1 | 1 | 0 | 0 | 2 |
| 76 | 1 | 0 | 0 | 0 | 1 |
| 77 | 0 | 1 | 0 | 0 | 1 |
| 78 | 0 | 2 | 0 | 0 | 2 |
| 79 | 0 | 2 | 0 | 0 | 2 |
| 81 | 0 | 1 | 0 | 0 | 1 |
| 82 | 2 | 1 | 0 | 0 | 3 |
| 83 | 0 | 1 | 0 | 0 | 1 |
| 86 | 1 | 2 | 0 | 0 | 3 |
| 87 | 0 | 1 | 0 | 0 | 1 |
| 93 | 0 | 2 | 0 | 0 | 2 |
| 94 | 1 | 1 | 0 | 0 | 2 |
| 96 | 0 | 1 | 0 | 0 | 1 |
| 97 | 0 | 1 | 0 | 0 | 1 |
| 100 | 0 | 1 | 0 | 0 | 1 |
| 102 | 0 | 1 | 0 | 0 | 1 |
| 108 | 0 | 1 | 0 | 0 | 1 |
| 112 | 1 | 0 | 0 | 0 | 1 |
| 113 | 0 | 1 | 0 | 0 | 1 |
| 118 | 0 | 1 | 0 | 0 | 1 |
| 121 | 0 | 1 | 0 | 0 | 1 |
| 128 | 0 | 1 | 0 | 0 | 1 |
| 133 | 0 | 1 | 0 | 0 | 1 |
| 137 | 0 | 1 | 0 | 0 | 1 |
| 145 | 1 | 1 | 0 | 0 | 2 |
| 151 | 0 | 1 | 0 | 0 | 1 |
| 157 | 0 | 1 | 0 | 0 | 1 |
| 159 | 0 | 1 | 0 | 0 | 1 |
| 174 | 1 | 0 | 0 | 0 | 1 |
| 183 | 0 | 1 | 0 | 0 | 1 |
| 194 | 1 | 1 | 0 | 0 | 2 |
| 207 | 1 | 0 | 0 | 0 | 1 |
| 209 | 0 | 1 | 0 | 0 | 1 |
| 214 | 1 | 0 | 0 | 0 | 1 |
| 228 | 0 | 1 | 0 | 0 | 1 |
| 230 | 0 | 1 | 0 | 0 | 1 |
| 237 | 1 | 0 | 0 | 0 | 1 |
| 239 | 1 | 0 | 0 | 0 | 1 |
| 248 | 1 | 0 | 0 | 0 | 1 |
| 249 | 1 | 0 | 0 | 0 | 1 |
| 250 | 1 | 0 | 0 | 0 | 1 |
| 270 | 1 | 0 | 0 | 0 | 1 |
| 292 | 1 | 0 | 0 | 0 | 1 |
| 296 | 1 | 1 | 0 | 0 | 2 |
| 306 | 0 | 1 | 0 | 0 | 1 |
| 374 | 0 | 1 | 0 | 0 | 1 |
| 424 | 0 | 1 | 0 | 0 | 1 |
| 11189 | 0 | 1 | 0 | 0 | 1 |
| 13039 | 0 | 1 | 0 | 0 | 1 |
| Total | 184 | 200 | 2 | 0 | 386 |

**Supplementary Table 7**. Tandem repeat characteristics (size/copy/type) in the mitochondrial genome of *C. medica*

| **NO.** | **Size** | **Copy** | **Repeat sequence** | **Percent Matches** | **Start** | **End** |
| --- | --- | --- | --- | --- | --- | --- |
| 1 | 18 | 2 | CAAAAATAAGTCAATATA | 100 | 35685 | 35720 |
| 2 | 32 | 2 | TCTGATCACTACGCTTTAGTGGCCAACGGGCG | 96 | 51150 | 51214 |
| 3 | 24 | 2 | AAAATAATAACACCATTAAAAAAT | 91 | 57748 | 57794 |
| 4 | 18 | 2.4 | CATAGTCGCGAGCTGTTT | 81 | 73704 | 73746 |
| 5 | 18 | 2 | ATCAGAATTTAGAGTTTC | 94 | 85432 | 85467 |
| 6 | 25 | 2 | TACAACTATCCACAAAGAAGCTAT | 87 | 92058 | 92105 |
| 7 | 40 | 2 | AAGATTCCAAACGGAATGAAAGCAGGAAATTGATTAGCTA | 100 | 122427 | 122506 |
| 8 | 16 | 2.3 | TGAGTTTTGGTTTGTT | 95 | 131910 | 131946 |
| 9 | 18 | 2.1 | AAGCAAGCCAAGCAATAGA | 90 | 151412 | 151449 |
| 10 | 8 | 4.2 | TAATTAAG | 92 | 154870 | 154903 |
| 11 | 13 | 1.9 | TAAACGAATCCCG | 100 | 199025 | 199049 |
| 12 | 18 | 2 | CTCAACCAATCTACTTAT | 94 | 229665 | 229700 |
| 13 | 16 | 2.3 | ACTCAAACAAACCAAA | 95 | 240612 | 240648 |
| 14 | 24 | 2 | TGTAACCCACGAAAGCTCGAGAAA | 100 | 243427 | 243474 |
| 15 | 16 | 2.3 | TGAGTTTTGGTTTGTT | 95 | 295156 | 295192 |
| 16 | 19 | 3.1 | TCTATTCTACGCTAATTCA | 89 | 300355 | 300412 |
| 17 | 18 | 2 | GAAACTCGAAATTCTGAT | 94 | 301742 | 301777 |
| 18 | 22 | 2.2 | TAGGGACTGGCAGGTAGTGCAGG | 85 | 324917 | 324965 |
| 19 | 18 | 2 | TGATGATAGTGACGATAT | 94 | 331263 | 331298 |
| 20 | 15 | 3.1 | AAACCCAAGGGTAAG | 88 | 338076 | 338123 |
| 21 | 16 | 3.1 | AAAACCCAAGGGTAAG | 88 | 338075 | 338123 |
| 22 | 18 | 2.4 | GACTATGAAACAGATCGC | 81 | 342822 | 342864 |
| 23 | 13 | 3.6 | AAGGAAAGGCAATA | 74 | 348727 | 348774 |
| 24 | 18 | 2.6 | GATCGACTGTGCGCAT | 82 | 377182 | 377224 |
| 25 | 24 | 3 | CAAATATCCAAGCTTTCTTTCCTC | 71 | 404634 | 404704 |
| 26 | 24 | 2.1 | CAGAAGATGAACCCGAGATCTCCC | 96 | 417557 | 417606 |
| 27 | 18 | 2.1 | TACTTAGATAGAAGAAGT | 100 | 456944 | 456980 |
| 28 | 23 | 2 | TAGAAGACTAAAGAAGAAAAGGG | 100 | 468947 | 468992 |
| 29 | 23 | 3 | TGTGGTACTTCATTATATGATAT | 100 | 481133 | 481201 |
| 30 | 5 | 5 | TTTAC | 100 | 509807 | 509831 |
| 31 | 28 | 2 | CTTAAGGGAAAGGGGCAAACAGGTCCTG | 96 | 530501 | 530556 |
| 32 | 13 | 2.9 | ATAATTTAATAAT | 92 | 551079 | 551115 |
